# Encoding Asymmetry of the N-Glycosylation Motif Facilitates Glycoprotein Evolution

**DOI:** 10.1371/journal.pone.0086088

**Published:** 2014-01-24

**Authors:** Ryan Williams, Xiangyuan Ma, Ryan K. Schott, Naveed Mohammad, Chi Yip Ho, Carey F. Li, Belinda S. W. Chang, Michael Demetriou, James W. Dennis

**Affiliations:** 1 Lunenfeld-Tanenbaum Research Institute, Mount Sinai Hospital, Toronto, Ontario, Canada; 2 Department of Molecular Genetics, University of Toronto, Toronto, Ontario, Canada; 3 Department of Laboratory Medicine and Pathology, University of Toronto, Toronto, Ontario, Canada; 4 Department of Ecology & Evolutionary, Cell & Systems Biology, University of Toronto, Toronto, Ontario, Canada; 5 Department of Neurology, University of California, Irvine, California, United States of America; 6 Department of Microbiology and Molecular Genetics, University of California, Irvine, California, United States of America; King’s College London, United Kingdom

## Abstract

Protein N-glycosylation is found in all domains of life and has a conserved role in glycoprotein folding and stability. In animals, glycoproteins transit through the Golgi where the N-glycans are trimmed and rebuilt with sequences that bind lectins, an innovation that greatly increases structural diversity and redundancy of glycoprotein-lectin interaction at the cell surface. Here we ask whether the natural tension between increasing diversity (glycan-protein interactions) and site multiplicity (backup and status quo) might be revealed by a phylogenic examination of glycoproteins and NXS/T(X≠P) N-glycosylation sites. Site loss is more likely by mutation at Asn encoded by two adenosine (A)-rich codons, while site gain is more probable by generating Ser or Thr downstream of an existing Asn. Thus mutations produce sites at novel positions more frequently than the reversal of recently lost sites, and therefore more paths though sequence space are made available to natural selection. An intra-species comparison of secretory and cytosolic proteins revealed a departure from equilibrium in sequences one-mutation-away from NXS/T and in (A) content, indicating strong selective pressures and exploration of N-glycosylation positions during vertebrate evolution. Furthermore, secretory proteins have evolved at rates proportional to N-glycosylation site number, indicating adaptive interactions between the N-glycans and underlying protein. Given the topology of the genetic code, mutation of (A) is more often nonsynonomous, and Lys, another target of many PTMs, is also encoded by two (A)-rich codons. An examination of acetyl-Lys sites in proteins indicated similar evolutionary dynamics, consistent with asymmetry of the target and recognition portions of modified sites. Our results suggest that encoding asymmetry is an ancient mechanism of evolvability that increases diversity and experimentation with PTM site positions. Strong selective pressures on PTMs may have contributed to the A+T→G+C shift in genome-wide nucleotide composition during metazoan radiation.

## Introduction

The structural and functional diversity of proteins is built on linear sequences of 4 nucleotides encoding the 20 amino acids, and hundreds of posttranslational modifications (PTMs), for an incredibly large molecular space. PTMs are often reversible, and they control protein localization, enzyme activities and information flow in the cell. Phylogenetic analysis of phosphopeptide sites has revealed that some sites are conserved, while many vary within unstructured regions of proteins [1]. Positional variation may reflect the evolution of partially redundant sites that mediate interactions within proteins and across protein networks [2]. Networks of partially redundant sites are likely to tolerate mutational loss and gain, more often than single-site PTMs that mediate high-affinity interactions. Evolvability is the capacity to generate heritable phenotypic variation, and depends on mechanisms that minimize the potential lethality of mutations such as partial redundancy, weak interactions and compartmentalization to increase mass action. Remarkable functionality arises from self-organizing molecular systems with these relaxed properties, which seem to challenge our instincts for symmetry and precision in everyday life. Evolvability also depends on poorly understood mechanisms that minimize the number of mutations needed to produce novel traits [3], and in this context we have examined the contribution of encoding asymmetry to genetic diversity in N-glycosylation sites and to the evolution of glycoproteins.

N-glycosylation has a number of features that are well suited for the analysis of PTM motifs and their impact on evolutionary rates. Secretory proteins are synthesized in the rough endoplasmic reticulum (ER) and modified on the lumen side of membrane by oligosaccharyltransferase (OST), which transfers the glycan from Glc_3_Man_9_GlcNAc_2_-pp-dolichol to Asn at NXS/T(X≠P) sites [4]. More than 97% of N-glycans are attached at NXS/T(X≠P) sites in mammalian glycoproteins, and substitution is highly specific for secretory proteins, allowing cytosolic proteins to serve as an internal control [5]. The mammalian STT3A and STT3B isoforms of OST act on flexible portions of pre and post folded proteins, respectively, in an efficient proof-reading system [6]. N-glycosylation is found in all domains of life [7], [8], and many secretory proteins are dependent on modification for chaperone- assisted folding. Protein homeostasis is an energy intensive process, and under stress conditions, reduced biosynthesis of Glc_3_Man_9_GlcNAc_2_-pp-dolichol can activate the unfolded protein response [9]. The ER chaperones calnexin/calreticulin bind to the Glc_1_Man_9_GlcNAc_2_-modification, and together with other heat-shock proteins (HSP), delay transit to the cell surface until folding is completed [10], [11]. As demonstrated for HSP90, chaperones allow protein variants to fold that might otherwise reduce fitness in current conditions, thereby buffering genetic diversity and increasing the probability of species survival during a severe environmental change [12].

The N-glycosylation motif is a product of very ancient selection for robustness in protein folding, thereby allowing functionality in more proteins sequences and survival of the genes encoding them. Moreover, ER N-glycans became a platform for Golgi remodeling and additional functionality at the cell surface; thus NXS/T underpins two levels of a hierarchy. The Golgi N-acetylglucosaminyltransferases Mgat1, −2, −4 and −5 initiate branches which are extended with sequences that act as ligands (epitopes) for a superfamily of carbohydrate-binding proteins or animal lectins [13]. The availability of receptors and solute transporters at the cell surface is regulated by N-glycan-lectin cross-linking, which slows diffusion in the membrane and loss to endocytosis (Fig. S1A–C) [14]. Tissue-specific expression of Golgi enzymes and metabolite flux into nucleotide-sugars determines the profile of remodeled N-glycans made in the Golgi. We have shown that galectin affinities for cytokine receptors are proportional to NXS/T number (heritable attachment sites) and to Golgi remodeling of N-glycans (conditionally regulated) [15]. Thus, the ER and Golgi levels of the hierarchy interact though the NXS/T attachment sites to regulate receptors and solute transporter at the cell surface. Golgi remodeling pathways emerged with metazoan radiation along with lectins [13], and a ∼5 fold increase in genes encoding secretory glycoproteins [16]. The expansion of genes families encoding glycoproteins such as morphogens and receptors were presumably under pressure to adapt rapidly during metazoan radiation [17].

The target and recognition portions of NXS/T are asymmetric for nonsynonomous and synonomous mutation, and as described herein, this asymmetry reveals evidence of recent selection and experimentation with N-glycosylation sites in vertebrate evolution. Moreover, genome-wide analysis and specific examples, suggests encoding asymmetry of NXS/T has contributed to evolutionary rates of glycoproteins. Other PTM motifs have similar attributes, as demonstrated herein for acetyl-Lys, which leads us to a wider discussion of encoding asymmetry as a foundational layer in the molecular hierarchy of evolvability itself.

## Results

### NXS/T Site Densities are Conserved in Secreted Proteins Despite Loss of Coding-potential

**Figure 1:**
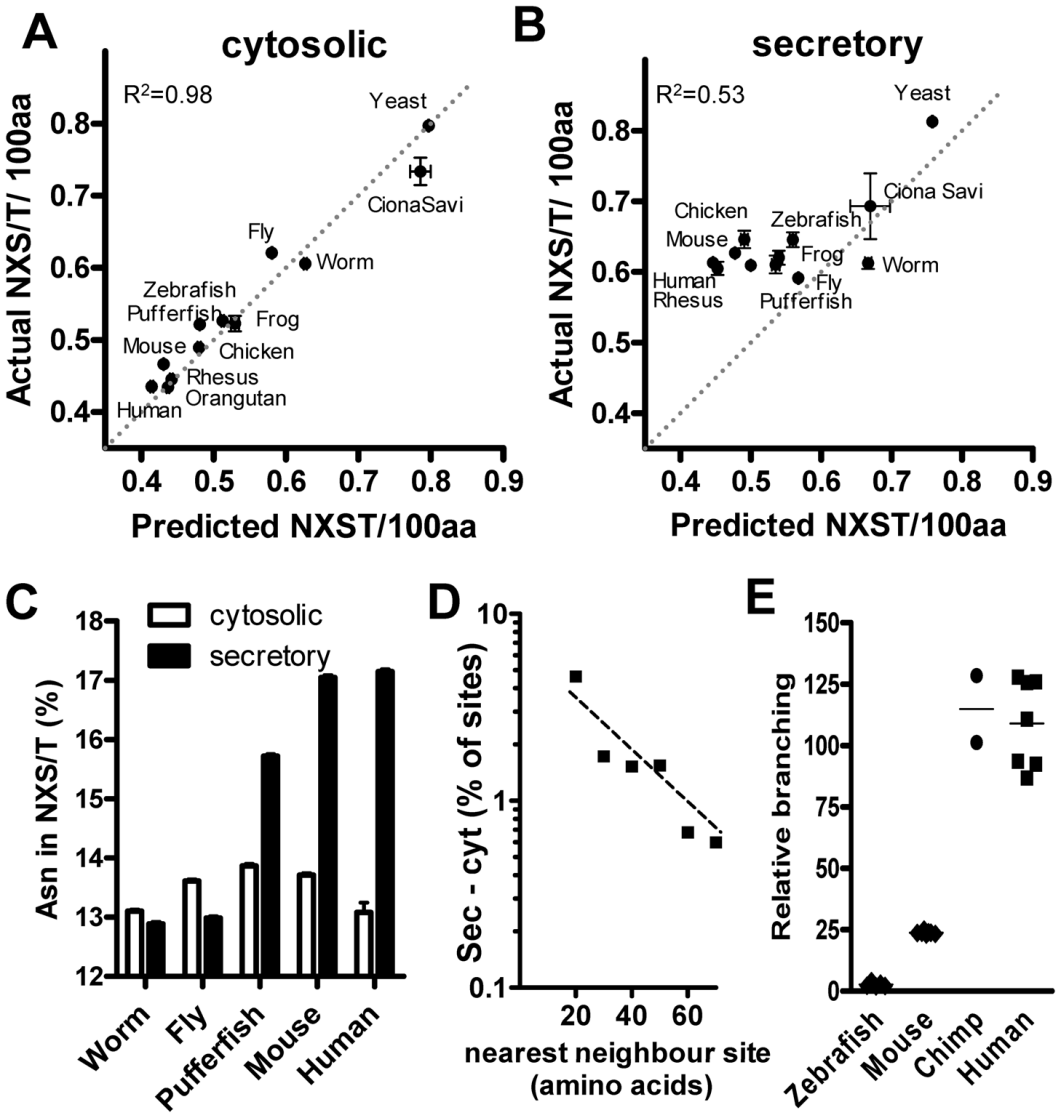
NXS/T site densities and genome compositions. (A,B) Actual NXS/T densities versus predicted densities from amino acid content in cytosolic and secretory proteins from species with high coverage genomes in Ensembl. Linearity analyzed by Pearson correlation. (C) Fraction of total Asn in NXS/T sites for each protein (mean ± SEM) in intracellular (cytosolic) and signal-sequence containing (secretory) proteins of five well annotated species. (D) NXS/T sites were binned by nearest neighboring, <20 then by increments of 10 amino acids. The difference between (sec) and cytosolic (cyt) bins bins reveals greater than expected proximity (skewness). The area under the line is ∼6.8% of all sites. (E) β1,6GlcNAc-branched N-glycans on lymphocytes from human, chimpanzee, C57/B6 strain mice, and zebra fish by L-PHA-FITC staining and FACS analysis. Each point is from an individual animal, except zebra fish where spleens were pooled.

Mutations are significantly biased in almost all animals in the direction G+C→A+T [18], while substitution favors the reverse, A+T→G+C [19], [20]. Substitution is the process where a germ line mutation becomes fixed in the population by either genetic drift or positive selection. The mechanism of mutation bias is unclear, but substitution bias in primates has been associated with hotspots of recombination, and may be due to errors in miss-match repair that have an A+T→G+C asymmetry [18]. On balance, there has been a progressive A+T→G+C shift in genome compositions with metazoan evolution, suggesting some form of selection with animal radiation is responsible for this imbalance (Fig. 1A). Of the amino acids comprising the N-glycosylation motif, Asn codons (AAC, AAT) are adenine “(A)”-rich, while Ser and Thr codons are near balanced for A, T, C but G-poor; therefore a net 1.6 times enrichment of A in the encoding of NXS/T (Fig. S2). The NXS/T density in cytosolic proteins declines with radiation from *Saccharomyces cerevisiae* to mammals in-step with declining A+T nucleotide content of the genomes (Fig. 1A). Cytosolic proteins are not targets of N-glycosylation and as expected, NXS/T densities drift with the genomic nucleotide content. In contrast, NXS/T densities in secretory proteins are conserved at ∼0.6 sites/100 amino acids comparing species from *Caenorhabditis elegans* to primates; this is despite a declining NXS/T coding potential reaching ∼30% in humans (Fig. 1B). As such, the proportion of total Asn in NXS/T sites of secretory proteins increases by 31% comparing worm to primates, consistent with increasing selective pressures on N-glycan functionality during metazoan radiation to primates (Fig. 1C).

It has been suggested that the decline in Tyr, an A+T rich encoded amino acid, may have been a result of site trimming; that is selection against interfering sites as various new tyrosine kinases evolved [21]. However, rather than simply trimming of deleterious sites, loss may create the conditions for selection of novel sites within a larger network of sites. The spacing of NXS/T sites in human secretory proteins is skewed towards clustering, which is consistent with selection for cooperative binding of N-glycans with lectins [22], [23] (Fig. 1D). The mammalian complement of branching enzymes (Mgat1, −2, −4 and −5) emerged in metazoans and has been present since a common ancestor with zebra fish (Fig. S1D,E). In search of more recent adaptations that may impact NXS/T positions, we measured branched N-glycan on lymphocytes and found very low levels on cells from zebra fish, intermediate from mice, and ∼ 4 fold higher on cells from chimpanzee and human (Fig. 1E). In vertebrates, three periods have been identified in the evolution of gene regulatory elements; early innovations near transcription factors and developmental genes, a trend that moved to extracellular signaling genes and then to PTM modifiers [24]. The latter and most recent innovations appear to include increased expression of branched N-glycans.

### Mutational Dynamics of NXS/T

**Figure 2:**
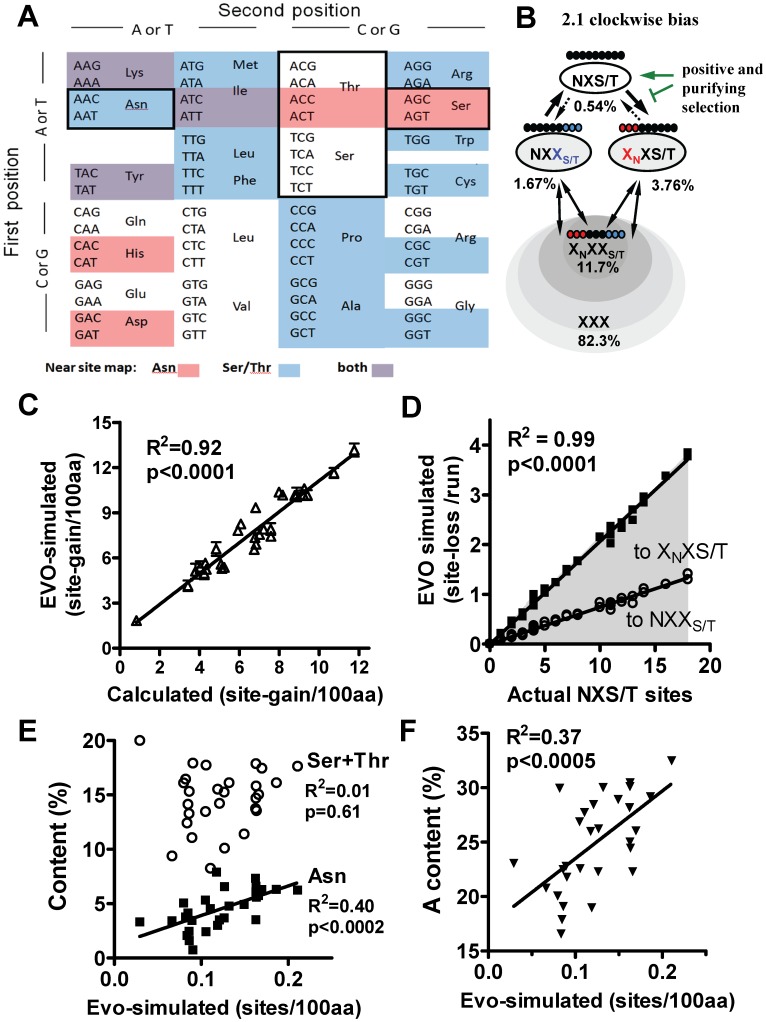
Encoding structure and simulations of NXS/T site gain and loss. (A) Codon chart highlighting the coding structure of NXS/T. Asn is A-rich with 14 neighbors (red), while 10 Ser/Thr codons have 31 codon neighbors (blue), for 89% and 53%, probabilities of nonsynonymous mutation, respectively. There are 64 codons, of which 61 encode amino acids and 3 encode a signal for termination of a peptide sequence. The 10,416 possible X_N_XS/T, NXX_S/T_ and NPS/T sequences are 3.01%, 1.59% and 0.03% of all possible coding 9-mer sequences (61^3^), respectively. (B) Asymmetry in the two major paths of loss and gain. The coding 9-mers indicated in the X_N_XS/T and NXX_S/T_ ovals are the subset of all coding 9-mer (% in brackets) are one mutation away from NXS/T. The solid arrows is the more likely pathway of interchange, while the broken arrows indicate the less probably pathway. The green arrows indicate how positive and purifying selection of NXS/T sites disrupts equilibrium in near-motifs. (C) The extracellular domains of 30 human secretory genes were serially mutated and subject to random selection used in the EvolveAGene 3.06 program (EVO-simulated, Y-axis). Simulated site gain in a set of human secretory proteins correlated with values calculated manually based on near-site numbers and paths into NXS/T (X-axis). (D) Site loss by EVO-simulation is proportional to actual sites in the initial sequences, and losses resulted in the expected ratio of X_N_XS/T to NXX_S/T_ near-sites. Site gain by EVO-simulation correlated with (E) Asn but not Ser+Thr content, (F) A nucleotide content.

Code usage and sign epistasis have been shown to constrain the possible evolutionary paths of bacterial genes under directional selection [25], [26]. Sign epistasis refers to positive and negative fitness effects of intermediate step leading to a selectable phenotype. For example, because of partial redundancy, site loss followed by gain at a novel position may be more likely than the reverse. Therefore NXS/T encoding asymmetry may facilitate repositioning (ie. positive sign epistasis), thereby promoting experimentation with novel site positions. Given the topology of the genetic code, most bipartite combinations of two amino acids are asymmetric for codon number and by extension, probable paths of loss and gain by mutation (Fig. 2A). However, if we consider the six codons for Ser and four for Thr as synonymous in the NXS/T motif, then the 1^st^ and 3^rd^ positions are even more asymmetric. NXS/T site loss is 1.7 times more likely by mutation of Asn than Ser/Thr, while site gain is 1.3 times more likely by generating a Ser or Thr downstream of an existing Asn (Fig. 2B). The value 1.7 multiplied by 1.3 = 2.1 reflects the asymmetry of creation and destruction of the motif by the two major paths. The asymmetry of conversion for all 20 by 20 simple bipartite motifs has a mean value of 1.28 (Fig. S3B,C). Therefore, mutational dynamics of NXS/T are due to both asymmetry of the genetic code and amino acid redundancy in the 3^rd^ codon position. Encoding asymmetry is based on codon number per amino acid. However, a critical observation is that asymmetry extends to the nucleotide level. Amino acid with fewer codons tend to be encoded by (A)-rich codons, thus mutation of (A) is more often a nonsynonomous event (Table S1).

To investigate site loss/gain dynamics, we performed simulated mutagenesis in the absence of selection on 30 human genes encoding glycoproteins. The data showed concordance between the simulated and calculated probabilities for site gain (Fig. 2C). The frequency of site loss was simply a function of the number of NXS/T sites present in the sequence. Loss of sites to X_N_XS/T was 1.7 times more frequent than to NXX_S/T_ as expected (Fig. 2D). The potential for site gain correlated with (A) and Asn content in coding and amino acid sequences, but not Ser+Thr (Fig. 2E,F). This is consistent with the encoding asymmetry where generating a Ser or Thr residue downstream of an existing Asn is the more probable path for site gain. In other words, site gain is more likely to be novel, than the reversal of a recently lost site. This describes the loss and gain dynamics of NXS/T in mechanical terms only, independent of selection.

To confirm the dynamics of NXS/T site, we identified primate gene homologues with a site gain or loss, where the direction of change from an ancestral allele could be determined (Table S2). Most of these changes are expected to be due to genetic drift and on average, directionally random [27]. We define X_N_ and X_S/T_ as amino acids one mutation away from Asn and Ser or Thr, respectively, and X_N_XS/T and NXX_S/T_ as “near-sites” one mutation away. As expected, loss of sites by mutation of Asn is ∼1.7 times greater than mutation of Ser/Thr, while gain of sites is 1.5 times more frequent from NXX_S/T_ than X_N_XS/T (Table S3). The change in NXS/T sites suggests a value for path asymmetry of 2.2 (calculated from the ratio of gain paths multiplied by the ratio of loss paths), which is close to the calculated value of 2.1 in neutral conditions (Fig. 2B).

### N-glycosylation Site Evolution Revealed by NXS/T Mutational Dynamics

**Figure 3:**
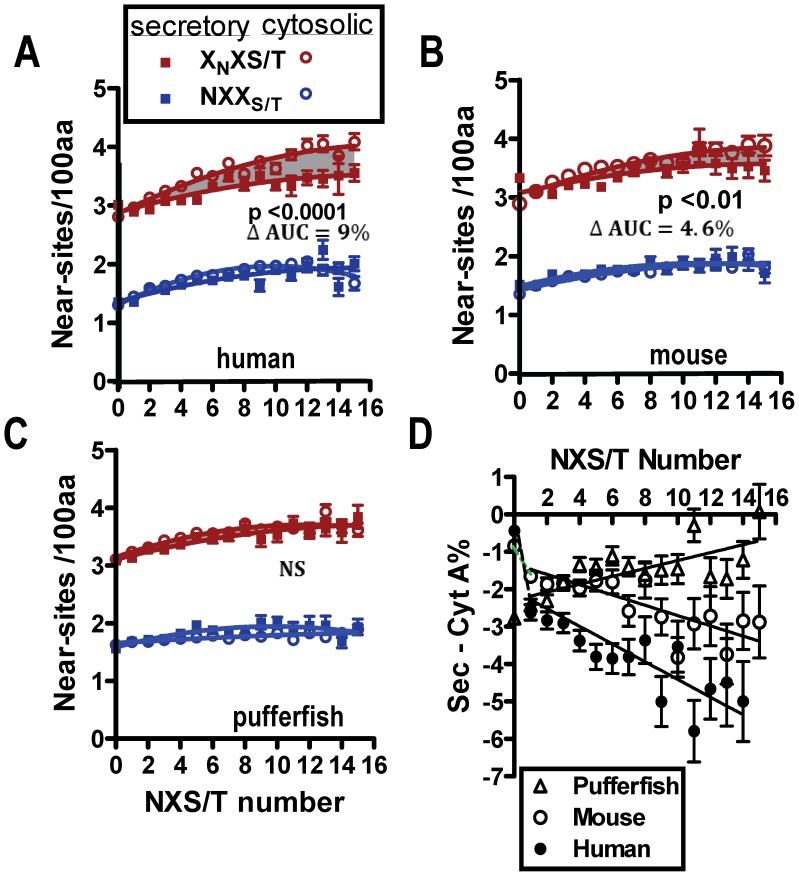
Selection of sites, and departure from neutrality in near-NXS/T and A nucleotide. (A–C) For human, mouse, and pufferfish, secretory and cytosolic proteins are grouped by actual NXS/T number, and near-sites were counted and expressed as density (mean/100 amino acids ±SE). X_N_XS/T (X≠P) densities of secretory and cytosolic proteins for human and mouse (grey shaded area) are significantly different by paired t-test. NPS/T represented only 0.03% of near-sites and was not significantly difference. Data for proteins with 0 to 15 sites is shown and represents 97.3% and 98.9% of all human sequences in secretory (n = 4440) and cytosolic (n = 15,626) proteins, respectively. (D) Difference in % (A) content for genes grouped by NXS/T number and expressed as secretory - cytosolic. Pearson correlation for mouse and human is p<0.0001. Note that a positive slope is expected in the absence of selection as exemplified by pufferfish.

Selection for N-glycosylation is indicated by increased NXS/T density and fraction of Asn found in the sites of secretory proteins with vertebrate evolution (Fig. 1C). We asked whether additional evidence of site selection might be found as a result of the neutral mutational cycle of NXS/T (Fig. 2B). Indeed, X_N_XS/T, the more likely product of site mutation, is reduced in human secretory proteins relative to cytosolic proteins (ie. cytosolic represents the null hypothesis) (Fig. 3A). X_N_XS/T depletion increases in proportion to the number and density of actual NXS/T sites in secretory proteins (Fig. 3A–C, S4A–D). The X_N_XS/T depletion was reduced in fish relative to primates. As NXS/T sites become fixed and no longer undergo neutral drift, X_N_XS/T becomes depleted. This is a deviation from equilibrium consistent with the effects of motif asymmetry and shifting selective pressures.

The NXS/T encoding potential in secretory proteins, along with (A) nucleotide content, declines in proportion to actual NXS/T number and with vertebrate evolution (Fig. 3D, S4E–H). Since sites densities (ie. 0.63 sites/100 amino acids) are not depleted over vertebrate evolution, it is possible that serial loss and gain has occurred under conditions of increasing selective pressure on sites. Thus (A) depletion may be a result of mutational loss at NXS/T sites, while X_N_XS/T depletion has resulted from positive selection of novel sites (scheme in Fig. 2B). With this interpretation of the data in Fig. 3A,D, positive selection for novel sites has occurred at rates that have exceeded replenishment of (A) and X_N_XS/T.

### NXS/T Sites Correlate with Evolutionary Rates of Secretory Protein

**Figure 4:**
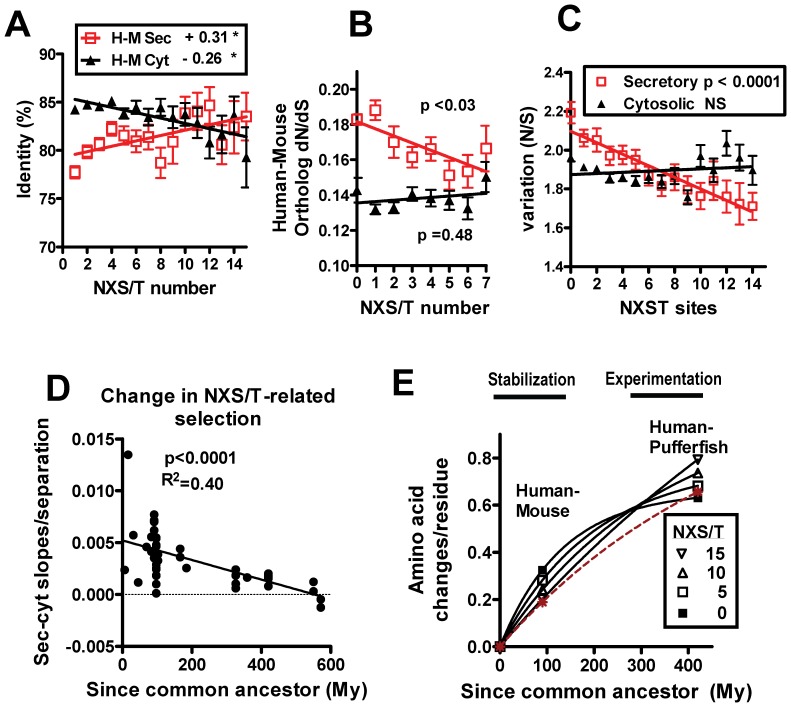
NXS/T multiplicity correlates with the evolutionary rates of secretory proteins. (A) Human-mouse and human-pufferfish amino acid identities for gene orthologues grouped by human NXS/T number, linear regression slopes are indicated, * Pearson correlation p<0.05. (B) Apparent evolutionary rates (*d*_N_/*d*_S_) of human-mouse orthologues grouped by site number. (C) Human SNPs in cytosolic and secretory proteins represented as *N/S*. (D) Slopes from NXS/T multiplicity versus amino acid identity (secreted-cytosolic)/separation time from human), were graphed for 52 animal species (Fig. S7)]. Pearson R^2^ = 0.40; Spearman correlation R = 0.696. (E) Amino acid substitution rates are proportional to NXS/T site number. The slopes from human-mouse and human-pufferfish % identity were used to calculate substitutions.

Secretory proteins are evolving faster than intracellular proteins in mammals, and this is independent of protein expression levels and protein-protein interactions [28]. One reason may be that secreted proteins are on average more recent innovations than cytosolic proteins, and therefore have had less time to evolve and reach relative stability [29]. However, when proteins are grouped by NXS/T multiplicity, we observed a positive correlation with human-mouse amino acid identity, and inversely with apparent evolutionary rates (alignment-wide *d*_N_/*d*_S_) (Fig. 4A,B). Cytosolic proteins are not N-glycosylated and serve as the intra-species reference for these analyses. We asked whether human polymorphisms (SNPs) might display a similar relationship with NXS/T multiplicity. Indeed, *N/S* decreases in proportion to NXS/T number in secretory but not cytosolic proteins, consistent with the trend in cross-species comparisons (Fig. 4C). The data suggests variation is more constrained with >6 NXS/T sites, and less constrained than neutral expectation with fewer sites. Human, mouse and rabbit are a similar distance from a common ancestor, but the mouse-rabbit comparison does not show a positive correlation between NXS/T number and sequence conservation (Fig. S5G). However, pairwise comparison of human to 52 species does suggest progressively increasing NXS/T-dependent selective pressure leading to human (Fig. 4D, S5). Viewed over vertebrate evolution, the average rate of amino acid change is proportional to NXS/T number in the human-pufferfish (fugu) comparison, whereas the relationship is inverted in the human-mouse comparisons. The inversion may be due to faster evolution ∼400 My ago in vertebrates glycoproteins in proportion to NXS/T number, which is reflected in the human-pufferfish comparison. This period of rapid evolution pre-dates the human-mouse comparisons, where glycoproteins are constrained (conservation) in proportion to NXS/T number (Fig. 4E). Thus N-glycosylation site number correlates with diversity and experimentation followed by earlier adaptation. N-glycans are polymers that have considerable reach on the surface of glycoproteins and so enhance epistasis though either intra-molecular interactions [30], or inter-molecular glycoprotein-lectin interactions [15].

### Acetyl-Lys Sites have Similar Properties

**Figure 5:**
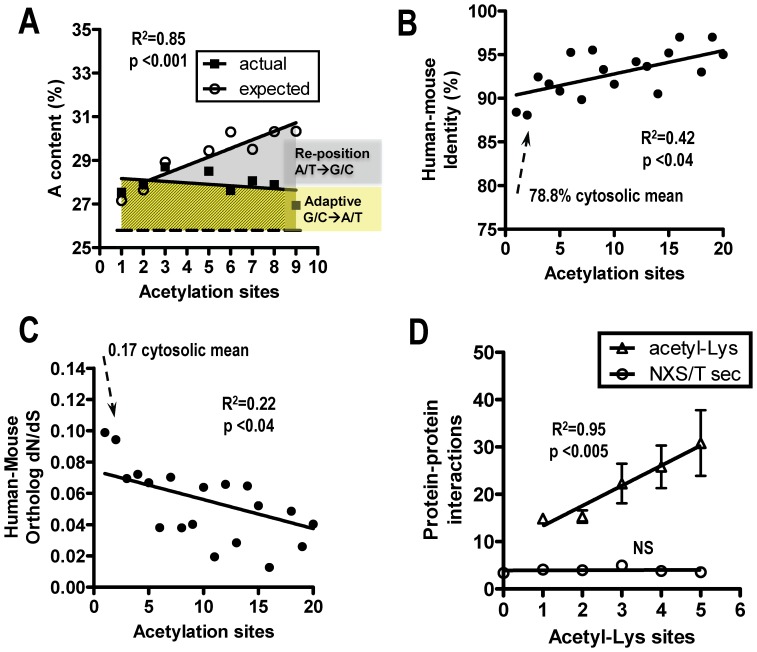
Acetyl-Lys site multiplicity and protein evolution. (A) human proteins (n = 2058) grouped by number of acetyl-Lys sites (4802 total sites) from ref [31]. A-nucleotide content in acetyl-Lys modified proteins is greater than expected (grey). The expected values are based on the regression slope for Lys content versus A-nucleotide content in all cytosolic proteins. Mean (A) content is 27.7% in acetyl-Lys modified proteins and 25.8% in non-modified cytosolic proteins (yellow) (P<0.01). Higher than average (A) content in acetyl-Lys modified proteins may reflect “sequence conditioning” due to acetyl-Lys experimentation and hitchhiking of adaptive sequences by linkage (B) human-mouse amino acid identity and (C) evolutionary rates (*d*_N_/*d*_S_) for orthologues grouped by site number. P value is for Pearson correlation. (D) Protein interactions from Biogrid (mean ±SE) grouped by number of acetyl-Lys sites, or NXS/T sites in secretory proteins.

If encoding asymmetry of NXS/T contributed to molecular evolutionary rates and shifting nucleotide composition, other PTMs are likely to have similar dynamics. Amino acids with similar physiochemical properties are in mutational proximity. Lys (AAA, AAG) is adjacent to Asn in the chart (Fig. 2B) and also has a reactive amino group that can undergo acetylation, methylation, ubiquitination as well as other modifications. Data from global identification of human acetyl-Lys sites by mass spectrometry [31], provides an opportunity to examine site experimentation, departure from neutrality in (A) content and effects of sites on sequence evolution. Many acetyl-Lys sites in proteins appear to be conserved [32], but the flanking linear sequences are considerably more degenerate than for N-glycosylation. Therefore, we compared acetyl-Lys site number to total Lys and (A) content in modified and non-modified proteins. Proteins with multiple acetyl-Lys sites display lower than expected (A) content, consistent with mutational dynamics where loss of sites is more likely by mutation of Lys than the accessory positions, similar to that observed for NXS/T (Fig. 5A). Data for proteins with multiple acetyl-Lys sites also displays evidence of sequence conditioning (ie. potential for novel sites), as well as sequence conditioning by site repositioning (Fig. 5A, yellow and grey, respectively). As observed for N-glycosylation sites, acetyl-Lys site number correlates with human-mouse sequence identity, and inversely with evolutionary rates (ie. *d*_N_/*d*_S_) (Fig. 5B,C).

The number of recorded protein-protein interactions increases with acetyl-Lys number, consistent with increasing stringent functional requirements and constrained evolutionary rates (Fig. 5D). Acetyl-Lys sites function as conditional surfaces for protein-protein interactions with bromodomain-containing proteins and possibly other interactions [33]. More generally, the number of protein interacting partners is reported to correlate with increased evolutionary constraint [34], whereas loss of shared interactions in duplicated gene pairs is associated with increased evolutionary rates [35]. NXS/T number also correlates with evolutionary constraint (Fig. 4A,B), but not with the recorded protein interactions (Fig. 5D). Glycoprotein - lectin interactions are qualitatively different, as there are redundancies and many possible interactions by bridging with lectins. This large set of weak interactions between membrane receptor and down-stream pathways increases the density of connections; which is an important feature of controllability in complex networks [36]. However, it is also possible that Biogrid has insufficient interaction data for secreted proteins.

### Site and Sequence Evolution in Paralogs

To explore the evolution of NXS/T sites in the context of specific genes, we examined the T cell co-receptors CD28 and CTLA-4, paralogs separated by ∼400 My, where site number is known to contribute to functionality [15]. CD28 and CTLA-4 mediate opposing effects on T cell proliferation by competing for the same counter-receptor, CD80/CD86 on antigen-presenting cells. NXS/T sites and near-sites are surface-exposed on human CD28 (5 sites) and CTLA-4 (2 sites), and distal from the binding site for the CD80/CD86 ligands (Fig. S6C,D). CTLA-4 has higher affinity for the counter-receptor, but depends on the two N-glycans sites and branching for surface residency [15]. In contrast, N-glycans on CD28 reduce the affinity for CD80/CD86, but surface levels CD28 are relative stable. [37]. During T cell activation, co-stimulation by CD28 and TCR promotes UDP-GlcNAc biosynthesis, which increases N-glycan branching on CTLA-4 and thereby surface retention by galectins [15]. A threshold level of surface CTLA-4 must be attained to block CD28 signaling and mediate T cell arrest. Primates have one less site in CTLA-4 and one more at N_37_ in CD28 compared to rodents. Fewer sites in CTLA-4 is consistent with an evolving trend toward a greater dependence on Golgi N-glycan branching and metabolism [38], [39]. Activity of the ancestral gene may have been CD28-like, as medaka and zebra fish have a single homologue, and experimental evidence suggests CTLA-4-like anti-proliferative activity is lacking [40].

**Figure 6:**
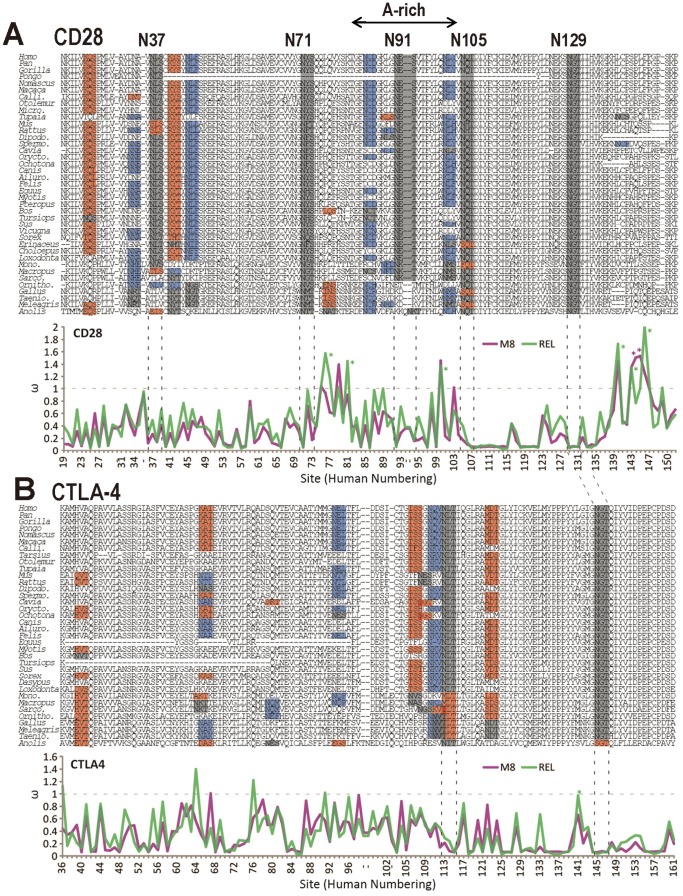
Variation in NXS/T sites and selective constraint across codons in CD28 and CTLA-4. Alignments for (A) CD28 and (B) CTLA4 along with line graphs depict the d*_N_*/d*_S_* ratio (*ω*) across sites as estimated by the M8 (PAML) and REL (HYPHY) models, for the extracellular domains of each gene. Glycosylation (NXS/T) sites are highlighted in black while X_N_XS/T and NXX_S/T_ near-sites are highlighted in red and blue, respectively. Vertical dashed black lines are used to indicate the position of highly conserved NXS/T sites on the line graph. The horizontal dashed grey lines indicate neutral evolution at *ω* = 1, where sites below and above this line are under negative and positive selection, respectively. Sites estimated to be at *ω* >1 with a posterior probability over 95% are indicated with an asterisk. Site numbering follows human, with sites absent in the human sequence (due to insertions in other sequences in the alignment) marked with dashes (−). The M8 and REL estimates for *ω* broadly agree. Overall, CD28 shows higher levels of *ω* and a greater number of NXS/T and near sites, as compared to CTLA4. An increase in *ω* is often observed flanking the NXS/T sequences (more so in CD28 than CTLA4). Abbreviations–***Calli.***, *Callithrix*; ***Micro.***, *Microcebus*; ***Dipodo.***, *Dipodomys*; ***Spermo.***, *Spermophilus*; ***Orycto.***, *Oryctolagus*; ***Ailuro.***, *Ailuropoda*; ***Mono.***, *Monodelphis*; ***Sarco.***, *Sarcophilus*; ***Ornitho.***, *Ornithorhynchus*; ***Taenio*** **.**, *Taeniopygia*.

Bayesian phylogenetic analyses [41] recovered gene trees for CD28 and CTLA4 genes that are generally consistent with the expected species topology (Fig. S6A,B). The phylogenies were utilized for analyses of selection using the random site models as implemented in PAML 4 [42], which estimate the *d*_N_/*d*_S_ ratio (*ω*) and the REL model of HYPHY, which allows for independent estimates of synonymous substitution rates (*d*_S_) [43]–[45] (Fig. 6, Tables S4A,B). A more detailed explanation of these methods can be found in the Methods section. Alignment-wide estimates of variation (*ω*) are high for both genes (M0, *ω* = 0.304 and 0.289 for CD28 and CTLA-4, respectively) compared to typical values of 0.08–0.18 [46]. The values for CD28 and CTLA-4 are comparable to genes coding highly diverse proteins with codon sites under strong positive selection, such as MHC proteins and reproductive proteins (*ω* = 0.5 and 0.27–0.93, respectively [47]. The proportion of codons in CTLA-4 under position selection is 0.5% compared to 5% in CD28 (Tables S4A,B). Overall, this indicates that CD28 is under less selective constraint and higher positive selection than CTLA-4, resulting in an increased rate of amino acid diversification, a trend that persists in primates (Fig. S6B).

Elevated *ω* values flank three of the five NXS/T sites in CD28 at N_71_, N_91_, N_105_, while values are lower in the homologous regions of CTLA-4, where two of the three sites are absent. The reduced selective constraint between N_71_ to N_105_ in CD28 is an (A)-rich sequence, and contains a higher density of near-sites (red or blue) aligning with an NXS/T (black) found in at least one species (Fig. 6A,B). The CD28 alignment displays a higher proportion of NXX_S/T_ (blue) to X_N_XS/T (red) compared to CTLA-4; thus a favorable gain to loss potential. The N_37_ site in CD28 is absent in most CTLA-4 homologues, but a proximal region of NXS/T experimentation marked by red and blue shows regionally higher *ω*. Conversely, NGT at N_129_ is conserved in both proteins (the only conserved site), and the proximal sequence also shows strong selective constraint. Thus six of seven NXS/T sites display evidence of repositioning coupled with regional relaxation of selective constraint, while one is conserved in all but one species and coupled with regional stability.

### Coding Asymmetry and Hitchhiking of Conditional Sequences

**Figure 7:**
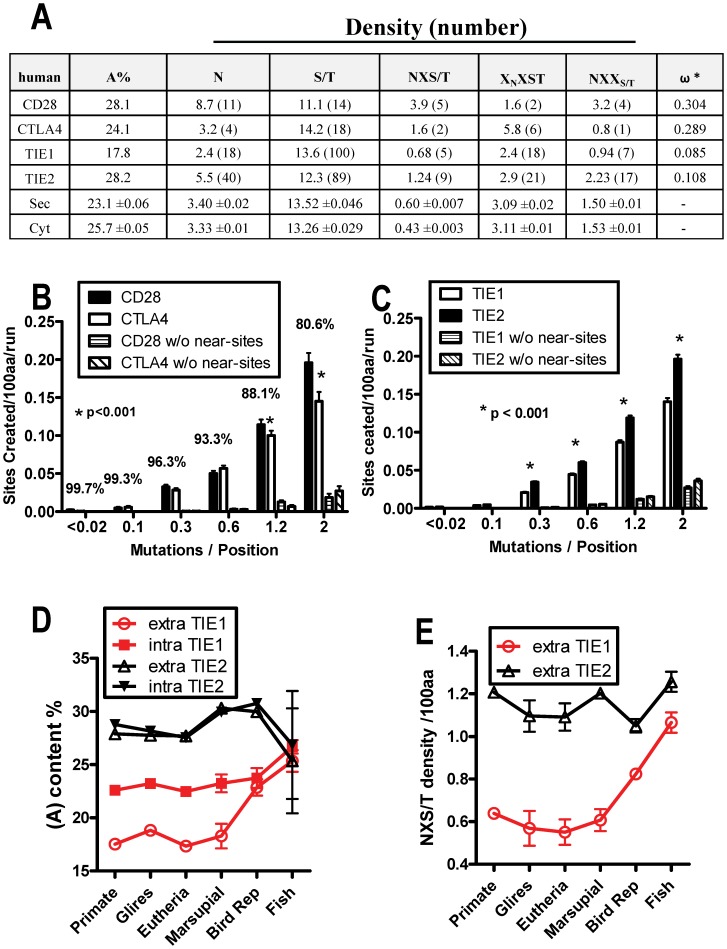
Site repositioning and hitchhiking of conditional neutral sequence. (A) Sequence compositions of CD28/CTLA-4 and TIE1/TIE2 paralogs, as well as the mean for secretory and cytosolic proteins. (B,C) The potential for site gain in the extracellular portions of the human genes was determined by simulated mutagenesis for CD28/CTLA-4 and TIE1/TIE2 paralogs. Sequences without near-sites (w/o) were created by replacement with GTA (Val) CCA (Pro) CTA (Leu), for five mutation steps away from NXS/T. Each bar is the mean ± SE of 10,000 runs with branch lengths in increments indicated by the upper limits on the X-axis (ie. mutations/position). (D) The (A) content in the intra- and extra- cellular portions of TIE1 and TIE2, grouping 53 species by clades. (E) NXS/T density comparing extracellular portions TIE1 and TIE2 by clades (Table S6).

Selective constraints were greater for the CTLA-4 sequence where directional selection has trimmed sites compared to CD28 and the ancestral gene. These observations suggest that directional selection for lower NXS/T site densities may condition the coding sequence for future change in the same direction. Conditionally neutral sequences or mutations do not alter phenotype, but may alter the fitness effects of subsequent mutations by epistasis [48]–[50]. Theory and data in bacterial models suggests that conditionally neutral substitutions accumulate more than random drift under selection by “hitchhiking” (ie. linkage with subsequently beneficial mutations), thereby reducing time-to-adaptation [48]–[50]. Here we consider near-NXS/T and Asn as conditional sequence that underpins the probability of NXS/T gain. The number of NXX_S/T_ near-sites, the more probable site for gain, is greater in CD28 than CTLA-4 (Fig. 7A), and simulated mutagenesis confirms that CD28 has greater potential for novel site gain (Fig. 7B). Thus CD28 and CTLA-4 have diverged not only in NXS/T number, but also the potential for novel site gain. In the context of site experimentation, selection for lower site number is expected to reduce (A), Asn and NXX_S/T_ content as observed in CTLA-4. Conversely, (A), Asn, and NXX_S/T_ in CD28 are enriched relative to CTLA-4, consistent with the direction of G+C→A+T mutational bias [18], and sequence conditioning that favors experimentation with novel sites.

A similar selection analysis as was performed for CD28 and CTLA-4 was also performed for the receptors TIE1 (5 sites) and TIE2 (9 sites) (Fig. S7A,B; Table S5A,B). The results from these analyses revealed that these receptors are an example of paralogs with near average *ω* levels (0.101 vs. 0.085 for TIE1; Table S5A,B). These *ω* values indicate significant rate variation in both genes (M3 vs M0, *P*<0.0001), but evidence for positive selection was not found in either dataset (M2a vs M1, M8a vs. M8, *P*>0.05; Table S4,S5). However, marked divergence between TIE and TIE2 for (A)-content and NXS/T number is readily observed during the divergence of fish/reptile, followed by stability in marsupials eutheria, glires and primates (Fig. 7D,E). Therefore, we implemented clade model C using PAML [51], along with a recently improved null model (M2a_rel) to test whether this evolutionary period correlated with differences in selective constraint [52]. The clade model C allows a class of codon sites to evolve at divergent rates among different partitions of the phylogenetic tree (Fig. S7A,B). Several sets of partitions were analyzed and compared, and the best fit for TIE1 divided therians (placentals and marsupials) and non-therians (in this case, birds, turtle, lizard, and platypus). For TIE2, the best-fitting partition divided eutherians (placentals) from non-eutherians (reptiles, monotreme, and marsupial). These models were found to be significantly better than the model that did not allow a divergent codon site class (M2a_rel, P<0.003) and had better likelihood scores than the other partitions tested (Table S6A,B) although the therian and eutherian partitions of TIE1 were very close. Therians and eutherians were found to have a lower ω with respect to the non-therians taxa (0.19 vs 0.25 and 0.016 vs 0.45) for TIE1 and TIE2. Subsequently, selective constraint and conservation of NXS/T sites in TIE1 and TIE2 has been strong in mammals. TIE2 retained higher NXS/T number (n = 9) and displays a higher early ω and greater decline in ω than TIE1 (n = 5) (Fig. S7A,B, Table S6A,B). This is consistent with the suggestion that N-glycosylation site number correlates with early experimentation that speeds adaptation of glycoproteins (Fig. 4E).

The (A) content of the extracellular portion of human TIE1 is ∼10% lower than TIE2, and shows ∼50% reduction in Asn (Fig. 7A). The direct loss of 4 NXS/T sites in TIE1 cannot account for this large a difference in (A) content. However, it is possible that site repositioning and loss in TIE1 has resulted in conditioning of the sequence by way of reduced (A) and Asn content (Fig. 7C). Moreover, the extracellular and intracellular homologies between human TIE1 and TIE2 are 30% and 76%, respectively, and only one of the NXS/T positions align between TIE1 and TIE2. Thus TIE1 and TIE2 display early NXS/T site experimentation, directional selection for site number, and conditioning of coding sequences, leading to selective constraint in mammals. Importantly, the intermediate level of (A) in the cytosolic portion of TIE1 suggests a bystander effect that is consistent with hitchhiking of adaptive sequences by linkage (Fig. 7D).

## Discussion

Here we describe a structural feature of the N-glycosylation motif that contributes to evolutionary experimentation with the position of N-glycan sites in secretory proteins. The codons for the target (Asn) and recognition amino acids (X-Ser/Thr) differ in their potential for synonymous and non-synonymous change, thus asymmetric paths to and from NXS/T sites. Based on principles of population genetics, many cycles of site gain and loss are possible over vertebrate evolution, but sampling falls far short of all possibilities. The asymmetry factor of NXS/T mutational dynamics (2.1 verses 1.28 average of all amino acid pairs) increases diversity in the population, and expands sampled-space exponentially over time. In permissive regions on the surface of the proteins, where NXS/T sites can reach densities similar to that of near-sites, encoding asymmetry contributes to diversity by promoting repositioning rather than reversal of a recently lost site. At each generation, more paths though sequence space are made available to natural selection.

We report that vertebrate evolution was accompanied by an increasing proportion of Asn in NXS/T sites of secretory proteins, and that site densities are conserved in the face of declining coding potential. Selective pressure on sites is revealed by intra-species deviation-from-equilibrium for secondary indicators; notably decrease in X_N_XS/T and (A) nucleotide content of secretory proteins relative to cytosolic proteins. These differences increase with site number per glycoprotein, and with radiation to primates. Taken together, we suggest that positive selection of sites disrupts the neutral NXS/T gain/loss cycle, and serial experimentation has resulted in the depletion of X_N_XS/T and (A) content. A phylogenetic examination of CD28/CTLA-4 and TIE1/TIE2 paralogs supports a role for motif asymmetry in site repositioning as well as conditioning the coding sequence for directional evolution toward high or low NXS/T site density. An intra-species comparison of secretory and cytosolic proteins indicates that N-glycosylation site number per glycoprotein correlates with evolutionary rates. As an example of other PTMs with similar encoding properties, the number of acetyl-Lys sites per protein also correlates with a departure from neutrality in (A) content, sequence conditioning and protein evolutionary rates.

It has been argued that selection against translation error and protein misfolding drives gene sequences toward a lower probability of nonsynonomous mutation and more efficient codon alternatives [53]. Grouping proteins by NXS/T number enriches for (A) content in a facile manner, and thereby increases the probability of nonsynonomous change. In keeping with the efficient codon hypothesis, cytosolic proteins grouped by NXS/T (i.e. (A)-enrichment) displayed decreasing sequence conservation, whereas, secretory proteins grouped the same way displayed increasing conservation (Fig. 4A,B). N-glycosylation promotes protein folding, which would be consistent with the efficient-folding hypothesis [53], but the positive correlation between NXS/T site number and sequence conservation runs counter to the efficient codon hypothesis. Therefore selection for N-glycan functions in ER and cell surface has had the greater influence on the evolution of secretory glycoproteins.

### Protein Evolutionary Rates

Epistasis is emerging as a major factor in molecular evolution rates, as most substitutions (90%) in the species where they occur, are estimated to be deleterious in a different background [54]. Many recently fixed substitutions in mammalian genes are estimated to be irreversible (ie. 10–40%), where a return to an ancient amino acid state would be detrimental in humans [55]. Therefore, step-wise repositioning of NXS/T sites may frequently involve directionality promoted by epistasis and gain/loss intermediates that become irreversible. For example, the human protease activated receptor-2 (F2RL1/PAR-2) has one NXS/T site at Asn222 which is required for normal surface levels of the pre-activated receptor. Human PAR-2 has lost sites at Asn23 and Asn30 that are present in chimp, gorilla, orangutan and marmoset. N-glycans at Asn23 and Asn30 markedly raise the threshold to protease cleavage at Ser34, which allows the clipped peptide to serve as the tethered ligand, activating the coagulation pathway [56]. Position 23 is polymorphic with Asn as a minor human allele (rs616235, 12%) suggesting a sequential change in Asn23 then Asn30 under directional selection. Site loss may have been selected for accelerated wound- healing, but may contribute to immune hypersensitivity in the modern human environment. As another example, the TGF-β family member Nodal lacks NXS/T sites and acts early in gastrulation over a few cell diameters, limited in distance by endocytosis and intracellular proteolysis. The experimental addition of NXS/T sites to Nodal positioned in the sequence analogous to those in BMPs 5,6 and 7 increased cytokine stability during recycling, thus the zone of Nodal signaling *in vivo* was enhanced 10 fold [57]. Changes in TGF-β/BMP activity in growth zones can effect rapid adaptation of beak size in isolated finch populations [58].

### Site Multiplicity and Cooperativity

Gain-of-NXS/T polymorphisms are more frequently associated with heritable human conditions than site loss [59]. This is consistent with the idea that site loss often buffers, whereas random placement of N-glycans has greater risk of disrupting the protein fold or altering function. However, multiple sites are not simply a buffer, but mediate cooperative binding that conveys important regulatory information in signal transduction pathways. For example, nine experimentally “movable” sites in the unstructured regions of *S. cerevisiae* SIC1 are progressively phosphorylated, and a threshold of six phosphorylated sites is required for CDC4 binding and G1/S progression [60]. The threshold number of six sites depends on the individual site on-off rates and affinities, as well as their spatial distribution. The amino acids flanking the phopho-Ser/Thr residue contribute to the affinity for CDC4. The interaction of CDC4 with multiple phopho-Ser/Thr sites in SIC1 is very dynamics, similar in this regard to N-glycan-lectin interactions that regulate glycoprotein dynamics at the cell surface [15], [61]. Mass-action is enhanced by co-localization of reactants, and therefore low-affinity multivalent interactions should promote experimentation and emergence of novel interactions [62].

The number and cumulative affinity of modified sites, and their spatial distribution on the surface of glycoproteins have adapted with changes in Golgi N-glycan remodeling and the expansion of genes encoding secretory proteins in animals. Receptor kinases with few NXS/T sites, require higher levels of N-glycan branching than receptors with five or more sites to generate affinity for galectins [15]. Receptors kinases that mediate growth signaling have significantly more N-glycosylation sites than receptors that mediate differentiation and arrest. The growth receptors display NXS/T densities exceeding the null hypothesis based on A/T content and amino acid content, consistent with their conservation. Growth signaling stimulates metabolism and increases UDP-GlcNAc and branching, which ultimately recruit low multiplicity receptors to the cell surface. For CD28/CTLA-4 and TIE1/TIE2, site number was reduced in one paralog and maintained in the other, while functions became mutually antagonistic. Site number in CTLA-4 is critical to T cell arrest, and a deficiency in sites contributes to autoimmunity [38]. Metabolism and branching of the two N-glycans on CTLA-4 increases with T cell activation, which promotes surface expression of CTLA-4 [15]. TIE1 and TIE2 are also antagonistic receptors in the regulation of angiogenesis. Angiopoietin-1 binds the endothelial TIE2 receptor and signals quiescence of endothelial cells while TIE1 forms a complex with TIE2 that inhibits signaling [63]. Like CTLA-4, the adaptive loss of four NXS/T sites in TIE1 may impose a greater dependence on UDP-GlcNAc metabolism and the Golgi branching pathways for cell surface expression.

### Asymmetries

As a reasonable expectation, the A+T→G+C substitution bias might be balanced by the countervailing asymmetry in mutations (G+C→A+T bias) [18]. However the former has dominated suggesting some form of selection that has driven down A/T content during vertebrate evolution. The A+T→G+C substitution bias favors mutation of Lys, Asn and Tyr codons, thereby loss of these PTM sites. Presumably site loss is often buffered by other sites, thus loss may be the catalyst or pre-condition for experimentation at novel positions. Site loss tends to deplete A/T content relative to gain in accordance with bipartite motif asymmetry. A+T→ G+C substitutions in primates were observed to be highly localized to genes [19], [20], and at a frequency in primates that suggests it should be detrimental to fitness. However, increasing N-glycosylation or acetyl-Lys site multiplicity correlated with the apparent repositioning of sites and faster evolutionary rates of the modified proteins. Therefore, the rapid evolution of these PTM systems may have outweighed the deleterious effects of A+T→G+C substitutions rates, and points to the importance of “creative destruction” as a pre-condition for novelty in PTM networks. This implies that stronger selective pressures on PTMs of A/T rich amino acids during vertebrate radiation may have tipped the balance between A+T→G+C substitution bias and mutations G+C→A+T bias. Indeed, a major portion of the proteome is either substrate, modifying enzyme and/or PTM-binding proteins for modifications to Lys, Asn and Tyr [64]. Therefore it possible that encoding asymmetries and selective pressures on PTMs targeting these amino acids may have contributed to shifting nucleotide content of genes and by extension genomes.

Motif encoding asymmetry is underpinned by the topology of the genetic code. Based on information theory, the topology of the genetic code minimizes the impact of translational error and the cost of information flow, while maximizing amino acid diversity. Tlusty [65] has suggested that evolution of the genetic code stopped short of the optimal chemical space, and mapping of up to 25 amino acids would have similar efficiency. However, such an increase would also reduce codon redundancy and thereby the effects of asymmetry as described herein. PTMs may have emerged in parallel with genetic code and contributed to the fixation of its topology. With these considerations, perhaps the topology of the genetic code became fixed at less than maximal encoding efficiency, due in part to orthogonal selective pressures on emerging PTM networks. Asn, Lys and Tyr have similar encoding properties, and their chemistries as PTM targets contribute to diversity. In addition, Tyr has physicochemical properties that enhance affinity and specificity at protein-protein interfaces, while serine and glycine (6 and 4 codons, respectively) act as spacers [66]. In this case, asymmetry plays that same role in repositioning the more important Tyr residues relative to spacers. Therefore the topology of the genetic code provides a specific measure of asymmetry (not too much or too little) that underpins evolvability in PTM networks and protein-protein interactions; pervasive and fundamental mechanisms of cellular organization.

## Methods

### Databases and Predictions of Motif Density

Genomic data was from the Biomart repository (http://www.biomart.org), and Ensembl. The longest transcript for each gene was analyzed at the nucleotide level for NXS/T sites and near-sites (1 mutational event away). Nucleotide and codon compositions of each coding sequence was determined with the software tool ACUA [67]. Signal sequences were predicted using SignalP [68]. Extra- and intracellular portions of transmembrane proteins were included in the secretory set, because automated predictions for membrane orientation are not reliable. Predictions were made using amino acid composition for each gene transcript or its nucleotide content weighted by mean transcriptome codon distribution. 4440 human secretory genes encode 16,044 NXS/T sites, for a mean frequency of 0.64±0.008 sites/100 amino acids. The expected values are 0.45±0.004 from amino acids, and 0.40±0.003 from nucleotide composition (p<0.0001).

### Motif Loss and Gain

Global analysis of N-glycans by mass spectrometry suggests a high stoichiometry of NXS/T occupancy regardless of Ser versus Thr in the 3^rd^ position [5]. N-glycosylation sites are 97% NXS/T in mice, and >75% of NXS/T sites are modified at high efficiencies. Therefore, Ser/Thr were considered as a block of 10 codons in the 3^rd^ position. The proportions of mutations that create, destroy and retain sites were calculated from the number of potential one-step mutations to, from and within Asn, Ser/Thr and their codon neighbors. For the purpose of comparing motif densities and near-sites across secretory and cytosolic proteins in different species, all mutations were assumed to have an equal chance of occurrence except those involving stop codons which were excluded. A motif is generated or lost due to ‘gain’ and ‘loss’ mutations, where there are two major paths to and from NXS/T. NPS/T is the 3^rd^ path and represents <2% of Δ(NXS/T) events based on its concentration in the transcriptome. Simulation by serial random mutations of 6 nucleotide (X_1_…X_2_) sequences with equal probability of nucleotide change produced the expected fractions of nonsynonymous events in NXS/T dynamics. For each of the 4096 (4^6^) possible nucleotide sequences for N…S/T, X_N_…S/T and N… X_S/T_ the ratios were 20∶68:140 respectively (a distribution equivalent to that seen in cytosolic proteins (0.44%, 1.53% and 3.08%). 17 of 31 X_S/T_ codons have two possible nucleotide changes to Ser/Thr, and 2 of 16 X_N_ codons have two paths to Asn. For all possible bipartisan motifs, paths of gain and loss were calculated using the proximity of codons (Fig. S3). Relative re-positioning rates for all pairs of amino acids combinations of the form X_1_…X_2_, expressed as the (ratio of gain paths)×(ratio of loss paths). For example, generation of FXP by one mutation can occur by 8 of 9 possible mutations (nonsynonomous) in the 1^st^ amino acid and 6/9 in 3^rd^ amino acid, therefore path ratios of 1.03 and 1.11 for an asymmetry value of 1.24. For example, out of 9 possible mutational events, Phe has eight possible pathways of loss and Pro has six. Therefore Phe is 8/6 times as likely to be destroyed (arrows out)., Tthe difference in rate of gain for Pro/Phe is 1.03/1.11 (See Fig. S3C). To obtain the asymmetry factor, we multiplied the gain and loss ratio to give FXP a repositioning value of 1.24. The median and mean for all combinations is 1.4 and 1.28, respectively.

### N-glycans Branching Measured by L-PHA Staining

For the experiment in Fig. 1E, human and chimpanzee peripheral blood mononuclear cells (PBMC) were isolated on Histopaque-1077™ (Sigma). Chimpanzee PBMC were purchased from Bioreclamation. Lymphocytes were prepared from C57/B6 mouse spleens as previously described [38]. Approvals were granted via human protocol 2001–2075 by UC Irvine Institutional Review Board and written informed consent was obtained for all human donors. The mouse protocol 2001–2305 was approved by UC Irvine Institutional Animal Care and Use Committee; and the zebra fish protocol by the Samuel Lunenfeld Research Institute Animal Care Committee. Samples were stained on ice for 30 minutes with 4 ug/ml of L-PHA-FITC (*Phaseolus Vulgaris* Leukoagglutinin; L-phytohemagglutinin) (eBioscience). MFI for L-PHA was determined on the L-PHA^+^ population by FACS, and species compared by normalization to human MFI. T cells from mouse and human were co-stained with anti-CD4 and L-PHA-FITC to detect the Mgat5 gene product [69]. Staining profiles showed a near normal distribution for all species.

### Simulation of NXS/T Coding Potential

To explore the potential in near-NXS/T sites, the EvolveAGene 3.05 algorithm was used to generate sequence diversity by mutagenesis and random selection of specific sequences [70]. The following settings were used: topology = symmetric; number of taxa = 2 (diverging input sequence into two); average branch length (equivalent to the mutagenesis events) was set at 0.02, 0.2, 0.3, 0.5, 1.0 and 2.5. Actual branch length for each simulation varied by random number generation; average selection on amino acid replacements = 0.016; selection against insertions and deletions = 0 (zero tolerance for indels); selection over sequences and branches = constant (no selection bias over specific region or branch); modifier = 1.0 (default). The output sequences were analyzed for creations and destructions of NXS/T sites, and branch length corresponding to these events. The cDNA sequence of the extracellular domain of human CD28, CTLA4, TIE1 and TIE2 were applied through the program. 10,000 simulations were performed for each gene. At branch length of one, sequences had ∼ 88% amino acid identity. The output sequences were analyzed for gain and loss of NXS/T sites. For the test set of 30 secretory proteins, we used the mature extracellular domains and 10,000 simulations at average branch length of 1.0. The genes were a set from the COSMIC database of somatic cancer mutations, where one or more ΔNXS/T was recorded, (gain is underline and loss is bold) ADAMTS16, BAI3, **CUBN**, ENPEP, **EPHA3,** **ERBB4, FGFR2,** FOLR2, GPC2, ISLR **KIT, LAMP1,** LGALS3BP, **MMP2,** NID1, PCDH7, PI15, PTGS2, WIF1. None of the somatic mutations were in the SNP data base for these genes. The stress response glycoproteins were added, ATF6-α, ATF6-β, CREB3, CREB3L1, CREB3L2, CREB3L4, CREBH, GLR IRE1 IRE2 and PERK, because N-glycosylation may regulate their activation, and be of interest for future functional studies.

### Phylogenetic and selection analyses

Coding sequences for CD28, CTLA-4, TIE1, and TIE2 from mammals and reptiles were obtained from ENSEMBL. BLAST searches, using the sequences from ENSEMBL, were used to check against the NCBI nucleotide database to ensure correct extraction of the coding sequence. Where mRNA and predicted mRNA sequences were available in the NCBI database, these were also used, either to replace ENSEMBL sequences or in addition to them. This resulted in 39 sequences for CD28, 36 for CTLA-4, 47 for TIE1, and 48 for TIE2. The sequences for each gene were aligned using codon alignment in MEGA 5 and then manually adjusted by eye. Gene trees were estimated in MrBayes 3 using reversible jump MCMC with a gamma rate parameter (nst = mixed, rates = gamma), which explores the parameter space for the nucleotide model and the phylogenetic tree simultaneously. Analyses were run for one million generations with a 25% burn-in and convergence was confirmed by checking that the standard deviations of split frequencies approached zero and that there was no obvious trend in the log likelihood plot. To estimate the form and strength of selection acting on the two genes, the alignments, along with the Bayesian gene trees, were analyzed with the codeml package of PAML 4 using random sites models (M0, M1a, M2a, M3, M7, M8a, and M8) [42]. In the context of these methods, “sites” are codons in the coding sequence.

Comparisons between the PAML random sites models were used to test for variation in *ω* (*d*_N_/*d*_S_) and for the presence of a positively selected class of sites. The PAML M0 model assumes all sites evolve under the same selective pressure, and estimates a single, average, *ω* value for the alignment. M1a assumes two classes of sites under purifying (0< *ω*_0_<1) and neutral selection (*ω*_1_ = 1), respectively, and is compared to M2a, which adds an additional class of sites under positive selection (*ω*_2_≥1). M3 relaxes the constraints placed on *ω*_0_, *ω*_1_, and *ω*_2_ allowing each to be estimated freely. M7 allows *ω* to continuously vary between 0 and 1 according to a beta distribution, and is compared to M8, which adds an additional class of sites under positive selection (*ω*_p_≥1). Model M8a, which restricts the additional site class to be neutral (*ω*_p_ = 1), was applied to test if the *ω* value estimated for the positively selected sites in M8 is significantly greater than one. All analyses were run starting with the branch lengths estimated by MrBayes repeated multiple times with varying initial starting points of *κ* (transition to transversion ratio) and *ω* to ensure convergence. The model pairs were compared using a likelihood ratio test (LRT) with a *χ* ^2^ distribution. Sites under positive selection in the M2a and M8 models were identified by the Bayes’ Empirical Bayes’ (BEB) analysis implemented in PAML [42].

Since PAML does not incorporate independently estimated rate variation in synonymous sites (*d*_S_), we also analyzed the data using the HYPHY [44] REL model [43] implemented on the Datamonkey webserver [45], which is similar to the PAML model, but allows variation in *d*_S_. The REL model defines X site classes, but does not implement LRT between a null and alternate model to test for the presence of a positively selected site class. Instead the model just estimates the ω value for each site in a comparable way to the BEB analysis of PAML [43].

Estimated *d*_N_/*d*_S_ (*ω*) values from the M8 and REL analyses were graphed for the extracellular domains of CD28 and CTLA-4 along with the alignments of each highlighting the NXS/T and near NXS/T sites. The REL model estimates for variation (*ω*) across codon sites largely agree with those of PAML M8, which confirms that an independently estimated *d*_S_ does not alter the results. In addition to the random sites models, clade model C (CmC) [51] was implemented for TIE1 and TIE2, along with a recently improved null model (M2a_rel) [52], in order to test for a class of divergently evolving sites. CmC assumes that some codon sites evolve conservatively across the phylogeny (two classes of sites where 0< *ω*_0_<1 and *ω*_1_ = 1), while a third class, the divergent site class, is free to evolve differently among two or more partitions (e.g., *ω*_2_>0 and *ω*_3_ ≠ *ω*_2_>0). Several partitions were tested including mammals vs. reptiles, placentals vs. non-placentals (reptiles, monotreme, marsupials), therians vs. non-therians, and primates vs. non-primates.

The PAML models revealed *ω*, the rate variation at each position for CD28 and CTLA-4 as expected for functional protein coding genes (M3 vs M0, *P*<0.00001) (Tables S4A,B). Significant evidence for positive selection (M2a vs M1, M7 vs. M8, *P*<0.001) was found in both genes, with the positively selected site class found to have an *ω* significantly greater than one (M8 vs. M8a, *P*<0.001; Tables S4A,B). For both CD28/CTLA-4 and TIE1/TIE2 paralogs, the REL model estimates for *ω* across codon sites largely agree with those of M8, which confirms that an independently estimated *d*_S_ does not alter the results.

#### Acetyl-Lys Sites

Acetylated-Lys sites verified by mass spectrometry as listed in table 1 of Choudhary et al [31], were grouped by site number, and the Lys and (A) nucleotide content taken from Ensembl V66. Human-human (9606–9606) protein-protein-interactions were obtained from Biogrid (BIOGRID-ALL-3.1.86). The proportionality of Lys and (A) content was compared for genes with and without acetyl-Lys sites.

#### Gene Homologues

The asymmetry of ΔNXS/T in gene homologues was analyzed for directionality (Table S2,S3). Human-chimpanzee one2one’ homologues from Ensembl (V 56) with near equal lengths ±15 nucleotides and without obvious insertion/deletion events were analyzed for ΔNXS/T. The direction of mutational events was determined from sequence alignments with four other primates. Sequence alignment was carried out with ClustalW 2.0 [71] and visualized with Figtree. Ribbons and surface representations were generated using PyMOL (DeLano Scientific). PDB IDs for the CD28, CTLA-4 and CTLA-4/CD80 complex correspond to 1YJD, 3OSK and 1I8L, respectively.

To model the effects of NXS/T number on amino acid sequence evolution, we let d_aa_ be the average fractional difference per position, and d_aa_ = 1-e^−Kaa^ where K_aa_ is the mean fraction of amino acid substitutions [72]. The mean change in d_aa_ for homologues with increasing NXS/T number is modeled as a linear relationship. The mean fractional substitution K_aa_ = -ln(1-d_aa_) was calculated for homologues grouped by human NXS/T number, for human-mouse and human-pufferfish gene comparisons, producing curvilinear relationships, consistent with NXS/T-dependent changes in evolutionary rates.

## Supporting Information

Figure S1 **N-glycosylation of proteins in the secretory pathway and emergence of N-glycan branching.** (A) Oligosaccharyltransferase (OST) transfers the glycan from Glc_3_Man_9_GlcNAc_2_-pp-dolichol to NXS/T sites in secretory proteins during translation in the endoplasmic reticulum (ER). The N-glycans promote protein folding either directly or with the aid of glycan-dependent chaperones calnexin, calreticulin and UGGT. Most glycoproteins transit through the Golgi en route to the cell surface, where N-glycans are variably remodeled and become ligands for animal lectins as well as pathogens. Functions at (1), (2) and (3) are regulated by multivalent interactions with lectin dependent on NXS/T site number and density as well as Golgi modifications. (B) Medial Golgi N-acetylglucosaminyltransferases (MGAT genes) initiate GlcNAc-branches. The complement of Mgat 1,2,4 and 5 were present in the common ancestor with fish. Fly and worm express a Golgi hexosaminadase that removes the Mgat1 product (dotted arrow) which prevents further N-glycan branching and extension. The absence of this enzyme in mammals, allows greater conditional regulation of branching by gene expression and UDP-GlcNAc levels. (C) Trans Golgi enzymes extend GlcNAc-branches, forming ligands for galectins, selectins and C-type lectins. (D) N-glycan structures change from yeast to chordates with the emergence of trimming and substitution to the in tri-mannosyl core, then change again in vertebrates with expanded use of the GlcNAc-branching enzymes. (E) Phylogenetic tree based on genomes, and marked in red to show the emergence of the Mgat branching enzymes. Mgat6 is only present in birds and a subset of fish.
(PDF)

Figure S2 **NXS/T coding potential correlates with genome nucleotide content.** The dotted lines are expected NXS/T density (neutral conditions) as a function of changing nucleotide compositions as indicated in the legend on the left. The symbols are expected NXS/T density based on the actual nucleotide compositions of secretory (sec) and cytosolic (cyt) transcriptomes as indicated in the legend on the right (Pearson R^2^ = 0.98, p<0.0001). The data points are near the theoretical line with a slope of 3.63, which is ∼1.61 times greater than for nucleotides used in equal proportion, notably 9/4 = 2.25.
(TIFF)

Figure S3 **Motif encoding structure and site loss-gain dynamics.** (A) Probabilities of loss and gain by mutation as a ratio for each amino acid. Those in red are A/T rich encoded. (B) A heat map of the amino acids in panel A showing all pairs of simple bipartite amino acids of the form X_1_…X_2,_ expressed as the (ratio of gain paths)×(ratio of loss paths) from the Table. (C) Weighting for each codon based on content in human secretory proteins, the table is arranged by codon number which correlates inversely with site-loss potential. Ratio of gain paths can be calculated from any two values in column 2 and ratio of loss paths can be calculated from any two values in column 3. Motif Asymmetry is expressed as (ratio of gain paths)×(ratio of loss paths).
(TIFF)

Figure S4 ***Disequilibrium in near-NXS/T and A-content.*** (A–D) Near-sites were counted in secretory and cytosolic proteins grouped by actual NXS/T number, and expressed as a density (mean/100 amino acids ±SE). X_N_XS/T (X≠P) densities of secretory and cytosolic for chimp and rat (grey shaded area) were significantly different by paired t-test. NPS/T represented only 0.03% of near-sites and did not show significant differences between secretory and cytosolic. (E–H) Expected NXS/T densities were calculated for each protein from amino acid (green) and nucleotide (red) compositions independently. The grey area is relative A nucleotide depletion.
(TIFF)

Figure S5 **Amino acid sequence identity of homologues grouped by NXS/T number.** (A) Human-mouse orthologues, grouped by human NXS/T number. The secretory proteins are a subset (n = 1822) of those in Fig. 4A which are confirmed for N-glycosylation by mass spectrometry (data from ref [16]). NXS/T in the sequences were used as site number on X-axis. *Pearson correlation slopes p<0.01. (B–E) Orthologues from species grouped into clades were compared to the human sequence. Note the increasing slope for secretory homologues from Ecdysozoa to Glires. (F) Each point is a slopes for secretory or cytosolic generated from human versus individual species primates (n = 10), glires (7), eutheria (18), marsupial (2), bird (3), fish (8), ciona (2), ecdysozoa (2) comparisons of one-to-one orthologues by NXS/T number. (G) Human, mouse and rabbit are a similar distance from a common ancestor. Unlike the human-mouse (Fig. 4A), the mouse-rabbit comparison does not show a positive correlation between NXS/T number and sequence conservation. (F) Each point is a slopes for secretory or cytosolic generated from human versus individual species primates (n = 10), glires (7), eutheria (18), marsupial (2), bird (3), fish (8), ciona (2), ecdysozoa (2) comparisons of one-to-one orthologues by NXS/T number. (G) Human, mouse and rabbit are a similar distance from a common ancestor. Unlike the human-mouse (Fig. 4A), the mouse-rabbit comparison does not show a positive correlation between NXS/T number and sequence conservation.
(TIFF)

Figure S6 **CD28 gene tree (A) and CTLA4 gene tree (B).** Estimated by Bayesian inference (MrBayes), and used for the PAML and HYPHY analyses in. Numbers at the nodes are posterior probabilities.
(PDF)

Figure S7 **TIE1 gene tree (A) and TIE2 gene tree (B).** Estimated by Bayesian inference (MrBayes), and used for the PAML and HYPHY analyses. The best fitting clade partition found in the clade model C (PAML) analyses is shown with reptiles and monotreme in blue and therian mammals in red. Numbers at the nodes are posterior probabilities. PAML and HYPHY analyses are in Table S6A.
(PDF)

Table S1 **Nonsynonymous and synonymous mutation sorted by nucleotides.** The effects of all nonsynonymous and synonymous mutation on nucleotide composition calculated, using the codon weighting of human genes encoding secretory proteins. Each mutational event has 9 possible outcomes per codon and characteristic probabilities of gain or loss via the four bases. Paths to stop codons have been excluded. Loss of A nucleotide with nonsynonomous changes is pronounced in the subset of amino acids with 2 codons, but the trend holds for the codon table as a whole. It should be noted that the sum of each row is zero, as expected for a substitution event.
(TIFF)

Table S2 **Chimpanzee-human homologues with gain or loss of NXS/T.** Summary of 63 gene homologues that differ in NXS/T site number, and direction of change could be determined by comparing with other primates. Sequences were used for analysis in Table S3.
(TIFF)

Table S3 **Asymmetry of change in NXS/T sites.** Gain or loss of NXS/T sites in 63 secreted human-chimpanzee homologues where the direction of change was indicated by comparison with homologues in other mammals. Path asymmetry is calculated from the ratio of site gains and losses (8/4.8×1.7/1.3) = 2.2. Precursor densities** were determined for this set of genes and nonsynonymous mutation rates* were based on codon table and human secreted composition. Expected and measured conversions are not significantly different by chi square contingency test. Colors correspond to Figure 2A, B.
(TIFF)

Table S4 **Results of random site PAML analysis on (A) CD28 and (B) CTLA-4.**
(TIF)

Table S5 **Results of random site PAML analysis on (A) TIE1 and (B) TIE2.**
(TIF)

Table S6 **Clade model C (PAML) analyses on TIE1 where the data was divided into multiple partitions (A) and clade model C (PAML) analyses on TIE2 where the data was divided into eutherian and non-eutherian partitions (B).**
(PDF)
