# Supplementary figures and images for: Encoding Asymmetry of the N-Glycosylation Motif Facilitates Glycoprotein Evolution
Source: PLoS One. 2014 Jan 24;9(1):e86088. doi: 10.1371/journal.pone.0086088 (PMC3901687; doi:10.1371/journal.pone.0086088)

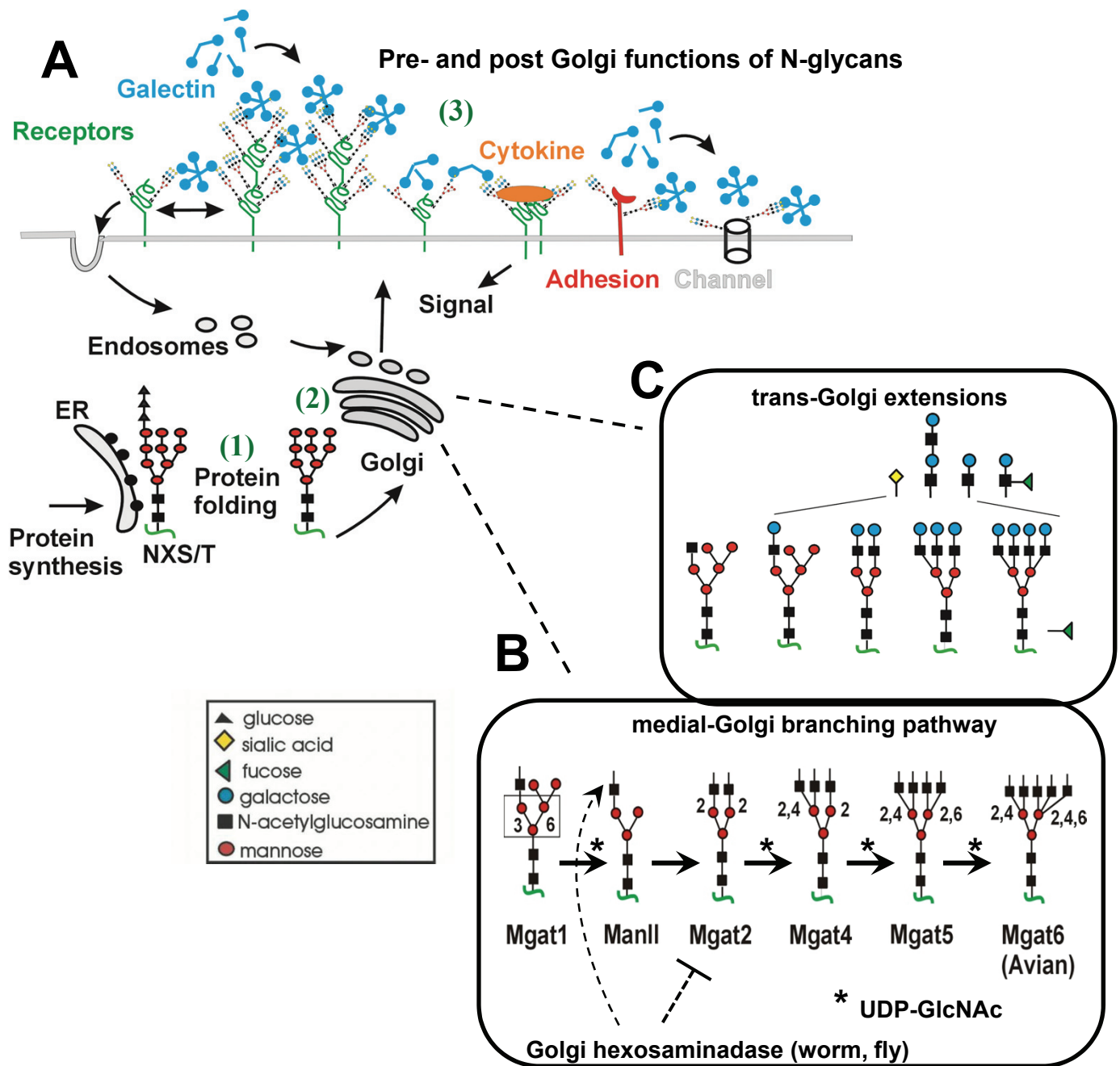

**D**

Vertebrates

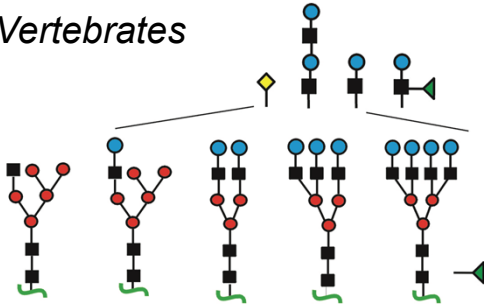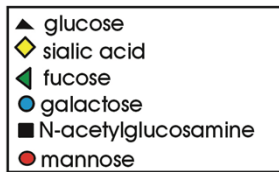

Worm, Fly

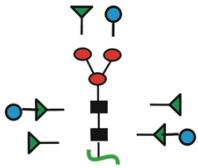

Yeast

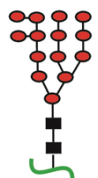

**E**

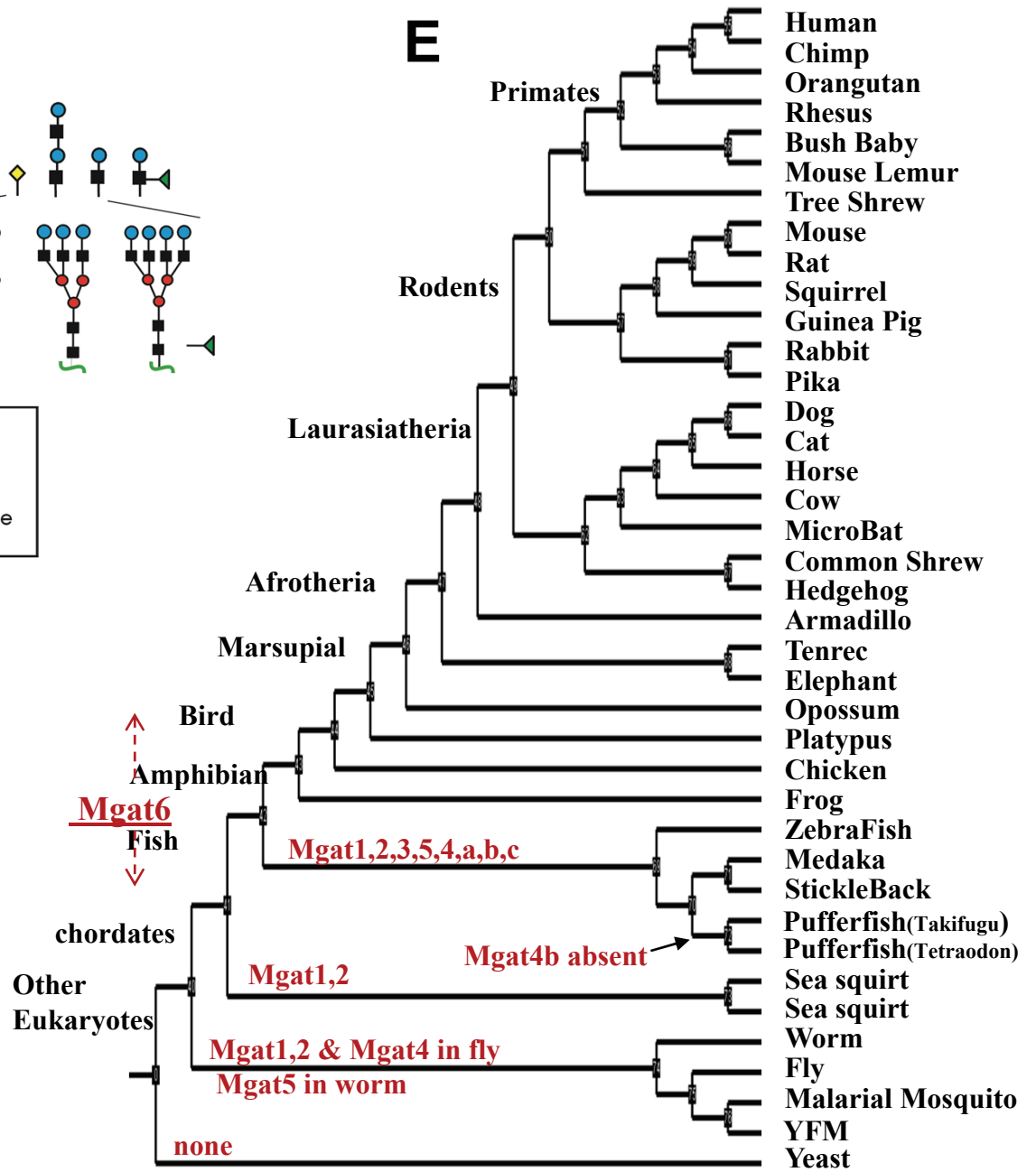

Supplement: Figure S1 — N-glycosylation of proteins in the secretory pathway and emergence of N-glycan branching. (A) Oligosaccharyltransferase (OST) transfers the glycan from Glc3Man9GlcNAc2-pp-dolichol to NXS/T sites in secretory proteins during translation in the endoplasmic reticulum (ER). The N-glycans promote protein folding either directly or with the aid of glycan-dependent chaperones calnexin, calreticulin and UGGT. Most glycoproteins transit through the Golgi en route to the cell surface, where N-glycans are variably remodeled and become ligands for animal lectins as well as pathogens. Functions at (1), (2) and (3) are regulated by multivalent interactions with lectin dependent on NXS/T site number and density as well as Golgi modifications. (B) Medial Golgi N-acetylglucosaminyltransferases (MGAT genes) initiate GlcNAc-branches. The complement of Mgat 1,2,4 and 5 were present in the common ancestor with fish. Fly and worm express a Golgi hexosaminadase that removes the Mgat1 product (dotted arrow) which prevents further N-glycan branching and extension. The absence of this enzyme in mammals, allows greater conditional regulation of branching by gene expression and UDP-GlcNAc levels. (C) Trans Golgi enzymes extend GlcNAc-branches, forming ligands for galectins, selectins and C-type lectins. (D) N-glycan structures change from yeast to chordates with the emergence of trimming and substitution to the in tri-mannosyl core, then change again in vertebrates with expanded use of the GlcNAc-branching enzymes. (E) Phylogenetic tree based on genomes, and marked in red to show the emergence of the Mgat branching enzymes. Mgat6 is only present in birds and a subset of fish. (PDF) [file pone.0086088.s001.pdf]

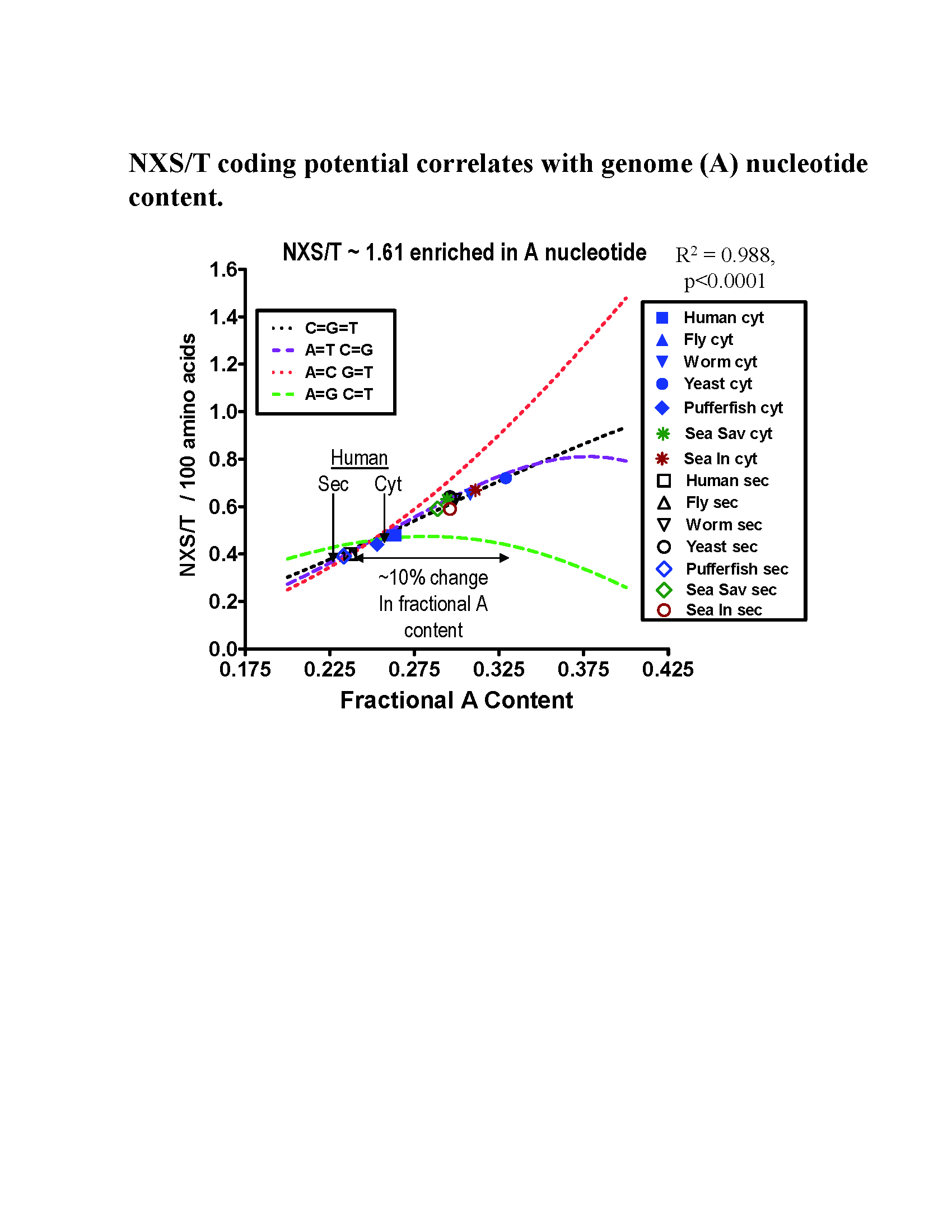

Supplement: Figure S2 — NXS/T coding potential correlates with genome nucleotide content. The dotted lines are expected NXS/T density (neutral conditions) as a function of changing nucleotide compositions as indicated in the legend on the left. The symbols are expected NXS/T density based on the actual nucleotide compositions of secretory (sec) and cytosolic (cyt) transcriptomes as indicated in the legend on the right (Pearson R2 = 0.98, p<0.0001). The data points are near the theoretical line with a slope of 3.63, which is ∼1.61 times greater than for nucleotides used in equal proportion, notably 9/4 = 2.25. (TIFF) [file pone.0086088.s002.tiff]

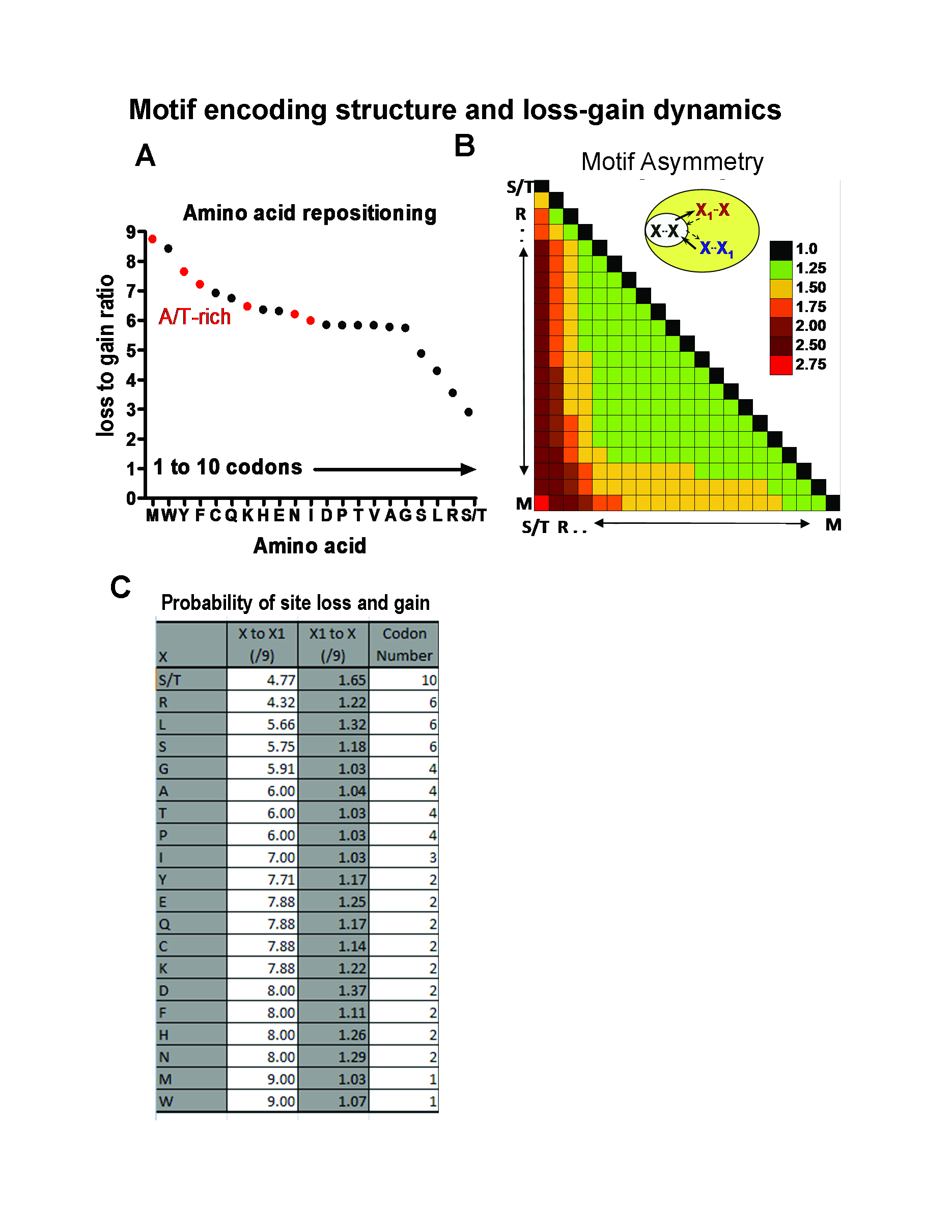

Supplement: Figure S3 — Motif encoding structure and site loss-gain dynamics. (A) Probabilities of loss and gain by mutation as a ratio for each amino acid. Those in red are A/T rich encoded. (B) A heat map of the amino acids in panel A showing all pairs of simple bipartite amino acids of the form X1…X2, expressed as the (ratio of gain paths)×(ratio of loss paths) from the Table. (C) Weighting for each codon based on content in human secretory proteins, the table is arranged by codon number which correlates inversely with site-loss potential. Ratio of gain paths can be calculated from any two values in column 2 and ratio of loss paths can be calculated from any two values in column 3. Motif Asymmetry is expressed as (ratio of gain paths)×(ratio of loss paths). (TIFF) [file pone.0086088.s003.tiff]

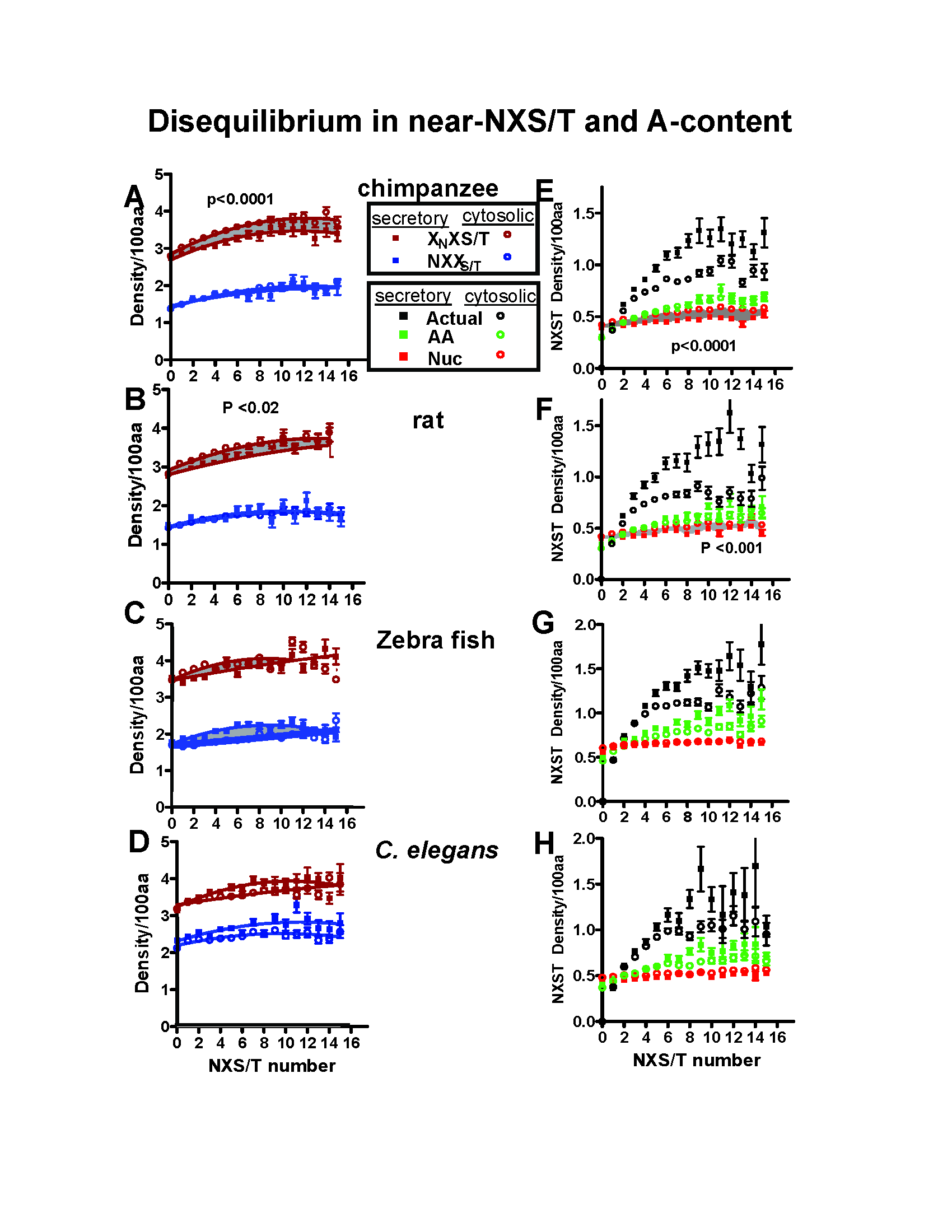

Supplement: Figure S4 — Disequilibrium in near-NXS/T and A-content. (A–D) Near-sites were counted in secretory and cytosolic proteins grouped by actual NXS/T number, and expressed as a density (mean/100 amino acids ±SE). XNXS/T (X≠P) densities of secretory and cytosolic for chimp and rat (grey shaded area) were significantly different by paired t-test. NPS/T represented only 0.03% of near-sites and did not show significant differences between secretory and cytosolic. (E–H) Expected NXS/T densities were calculated for each protein from amino acid (green) and nucleotide (red) compositions independently. The grey area is relative A nucleotide depletion. (TIFF) [file pone.0086088.s004.tiff]

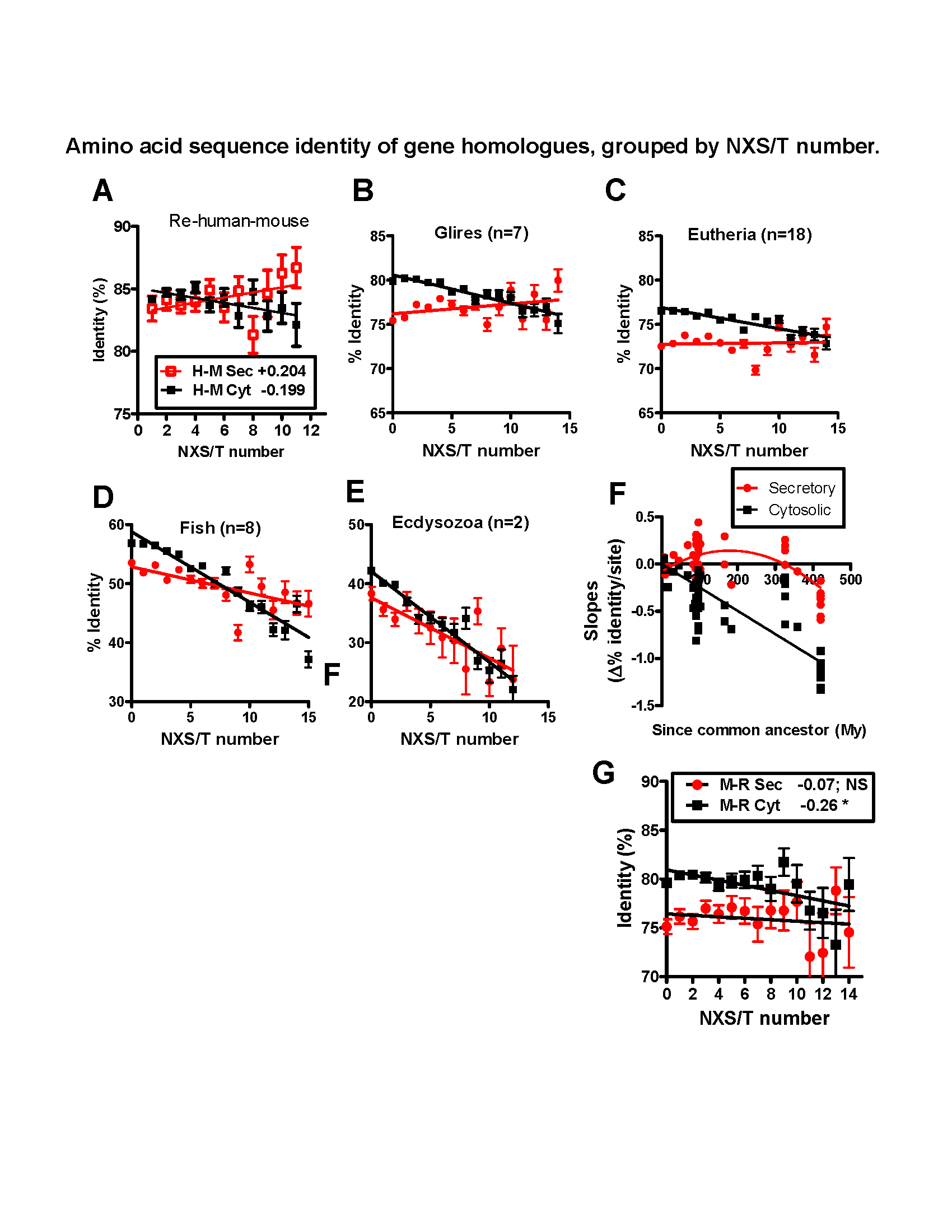

Supplement: Figure S5 — Amino acid sequence identity of homologues grouped by NXS/T number. (A) Human-mouse orthologues, grouped by human NXS/T number. The secretory proteins are a subset (n = 1822) of those in Fig. 4A which are confirmed for N-glycosylation by mass spectrometry (data from ref [16]). NXS/T in the sequences were used as site number on X-axis. *Pearson correlation slopes p<0.01. (B–E) Orthologues from species grouped into clades were compared to the human sequence. Note the increasing slope for secretory homologues from Ecdysozoa to Glires. (F) Each point is a slopes for secretory or cytosolic generated from human versus individual species primates (n = 10), glires (7), eutheria (18), marsupial (2), bird (3), fish (8), ciona (2), ecdysozoa (2) comparisons of one-to-one orthologues by NXS/T number. (G) Human, mouse and rabbit are a similar distance from a common ancestor. Unlike the human-mouse (Fig. 4A), the mouse-rabbit comparison does not show a positive correlation between NXS/T number and sequence conservation. (F) Each point is a slopes for secretory or cytosolic generated from human versus individual species primates (n = 10), glires (7), eutheria (18), marsupial (2), bird (3), fish (8), ciona (2), ecdysozoa (2) comparisons of one-to-one orthologues by NXS/T number. (G) Human, mouse and rabbit are a similar distance from a common ancestor. Unlike the human-mouse (Fig. 4A), the mouse-rabbit comparison does not show a positive correlation between NXS/T number and sequence conservation. (TIFF) [file pone.0086088.s005.tiff]

A

# TIE1 gene tree

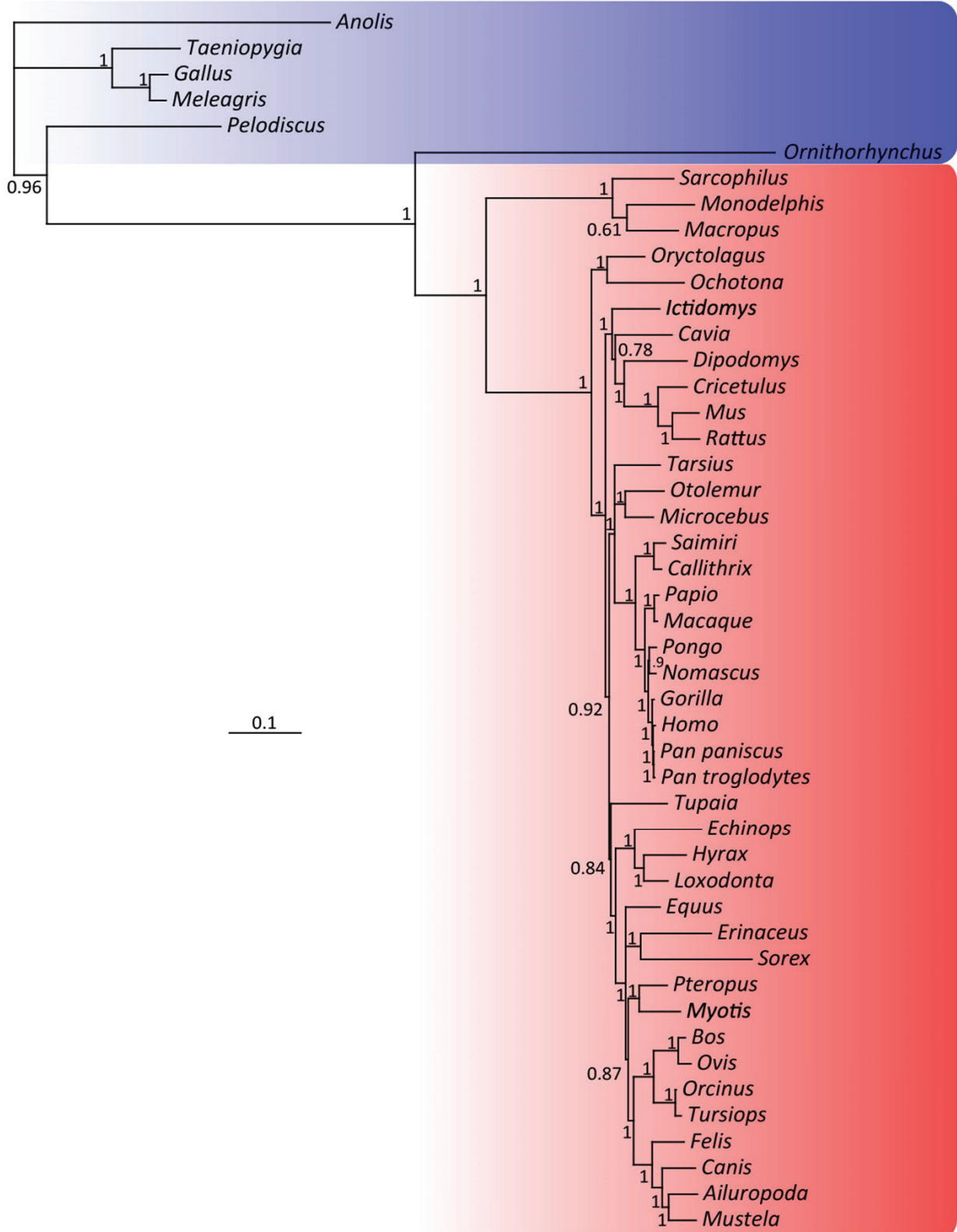

**B**

## TIE2 gene tree

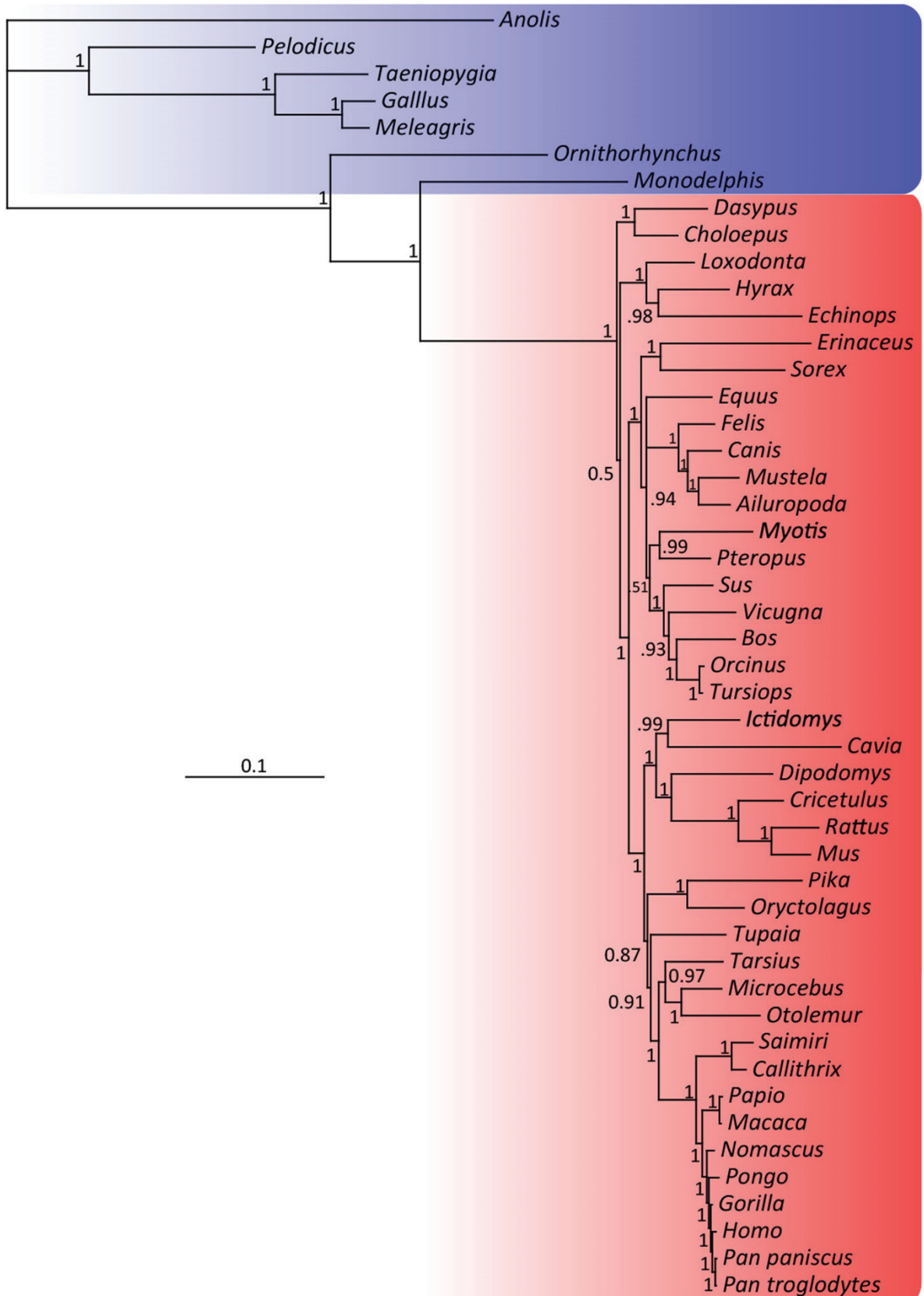

Supplement: Figure S7 — TIE1 gene tree (A) and TIE2 gene tree (B). Estimated by Bayesian inference (MrBayes), and used for the PAML and HYPHY analyses. The best fitting clade partition found in the clade model C (PAML) analyses is shown with reptiles and monotreme in blue and therian mammals in red. Numbers at the nodes are posterior probabilities. PAML and HYPHY analyses are in Table S6A. (PDF) [file pone.0086088.s007.pdf]

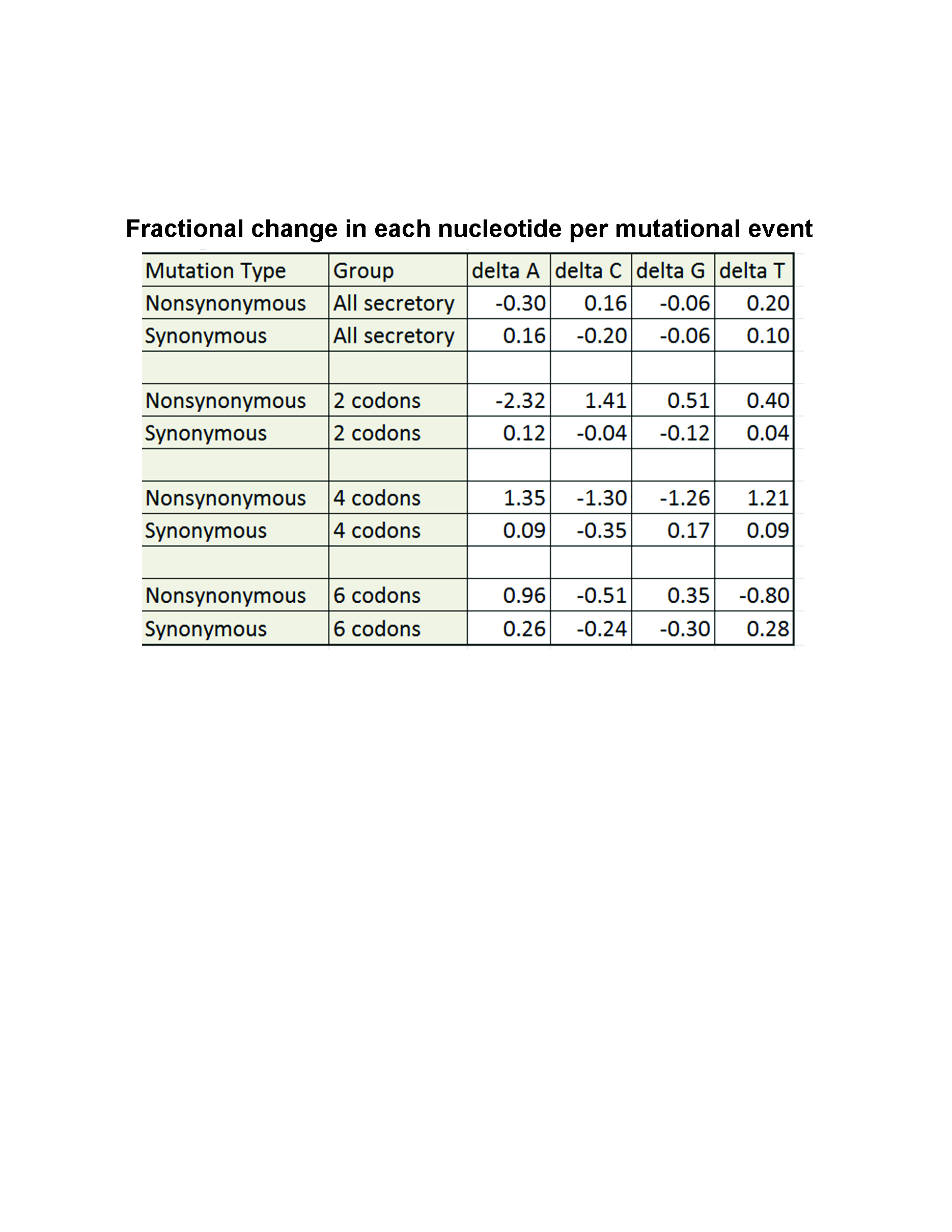

Supplement: Table S1 — Nonsynonymous and synonymous mutation sorted by nucleotides. The effects of all nonsynonymous and synonymous mutation on nucleotide composition calculated, using the codon weighting of human genes encoding secretory proteins. Each mutational event has 9 possible outcomes per codon and characteristic probabilities of gain or loss via the four bases. Paths to stop codons have been excluded. Loss of A nucleotide with nonsynonomous changes is pronounced in the subset of amino acids with 2 codons, but the trend holds for the codon table as a whole. It should be noted that the sum of each row is zero, as expected for a substitution event. (TIFF) [file pone.0086088.s008.tiff]

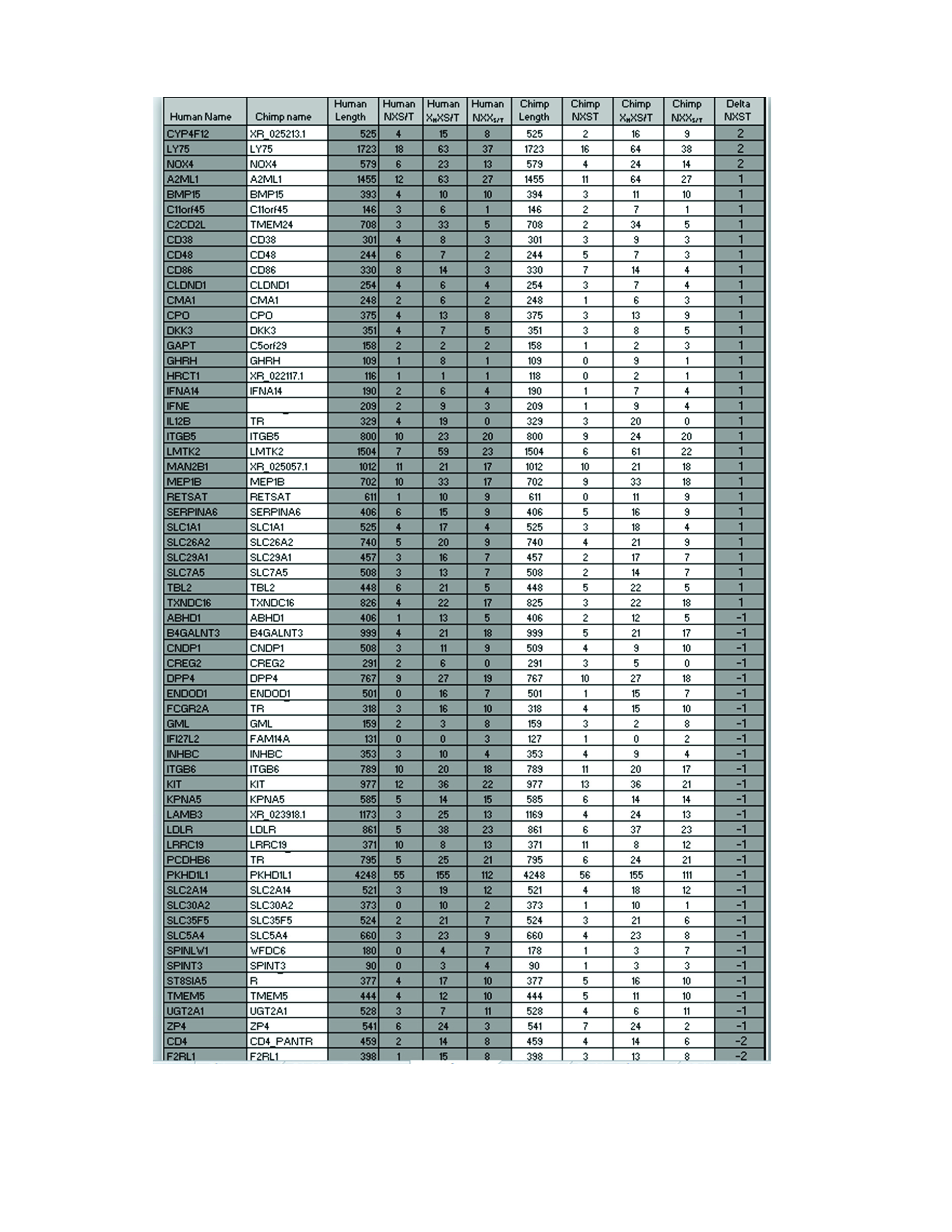

Supplement: Table S2 — Chimpanzee-human homologues with gain or loss of NXS/T. Summary of 63 gene homologues that differ in NXS/T site number, and direction of change could be determined by comparing with other primates. Sequences were used for analysis in Table S3. (TIFF) [file pone.0086088.s009.tiff]

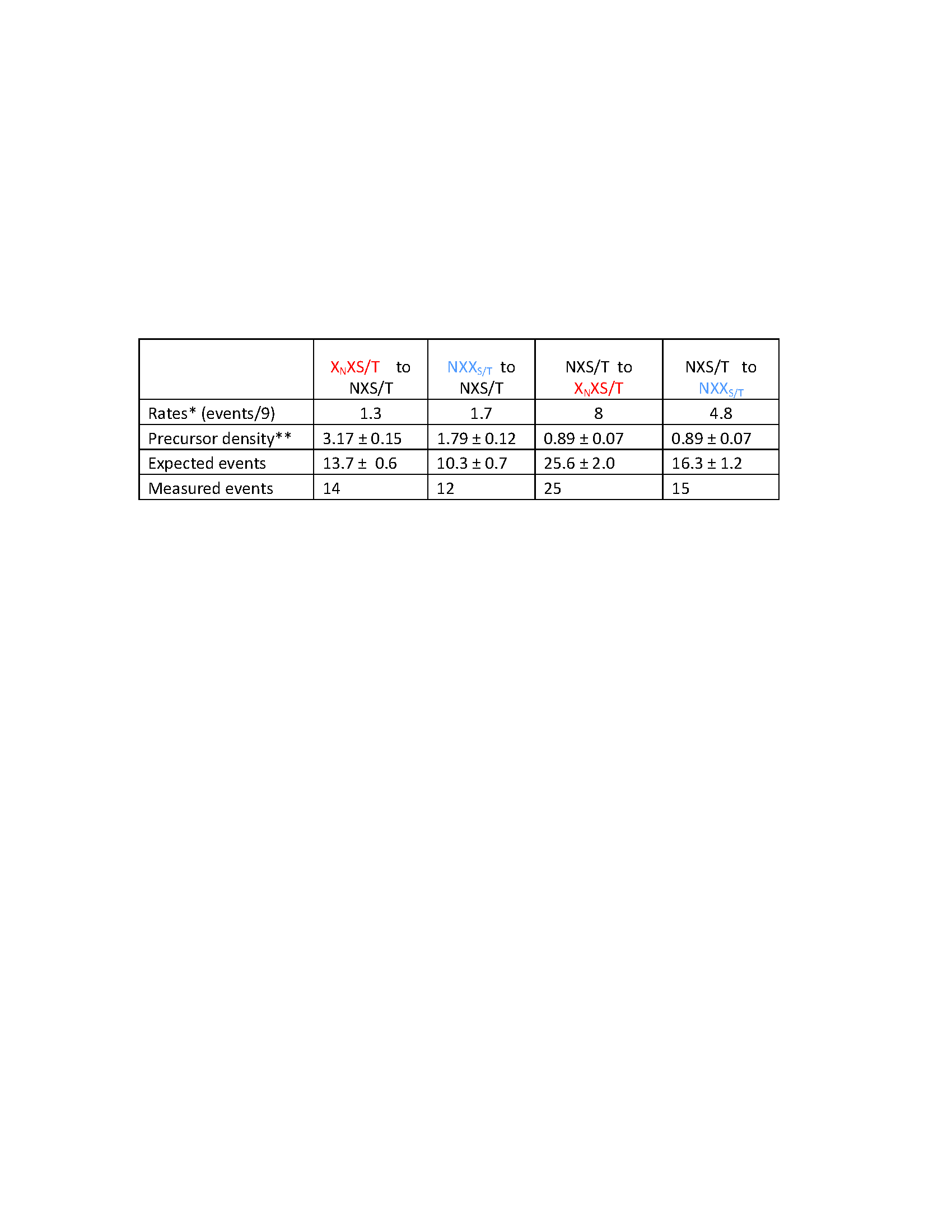

Supplement: Table S3 — Asymmetry of change in NXS/T sites. Gain or loss of NXS/T sites in 63 secreted human-chimpanzee homologues where the direction of change was indicated by comparison with homologues in other mammals. Path asymmetry is calculated from the ratio of site gains and losses (8/4.8×1.7/1.3) = 2.2. Precursor densities** were determined for this set of genes and nonsynonymous mutation rates* were based on codon table and human secreted composition. Expected and measured conversions are not significantly different by chi square contingency test. Colors correspond to Figure 2A, B. (TIFF) [file pone.0086088.s010.tiff]

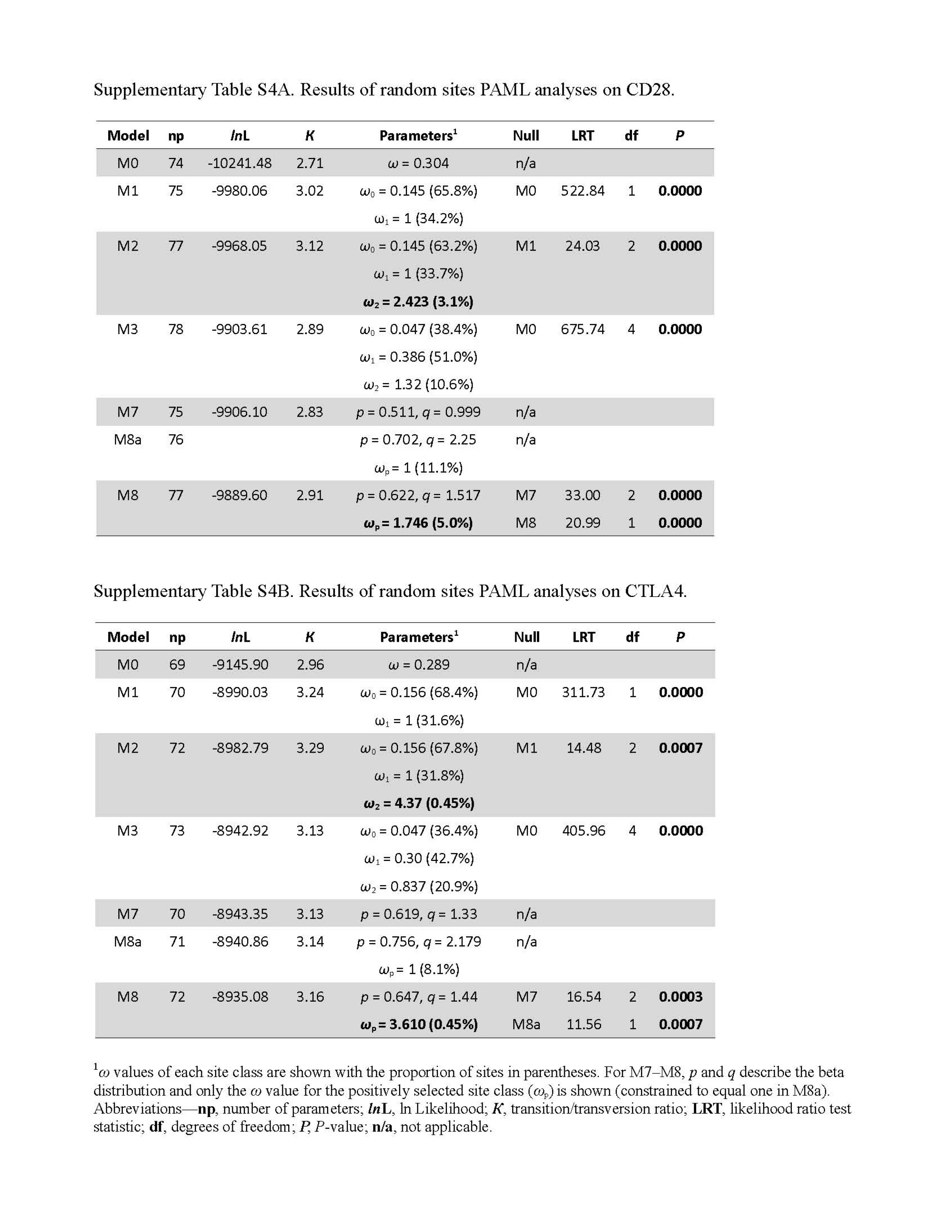

Supplement: Table S4 — Results of random site PAML analysis on (A) CD28 and (B) CTLA-4. (TIF) [file pone.0086088.s011.tif]

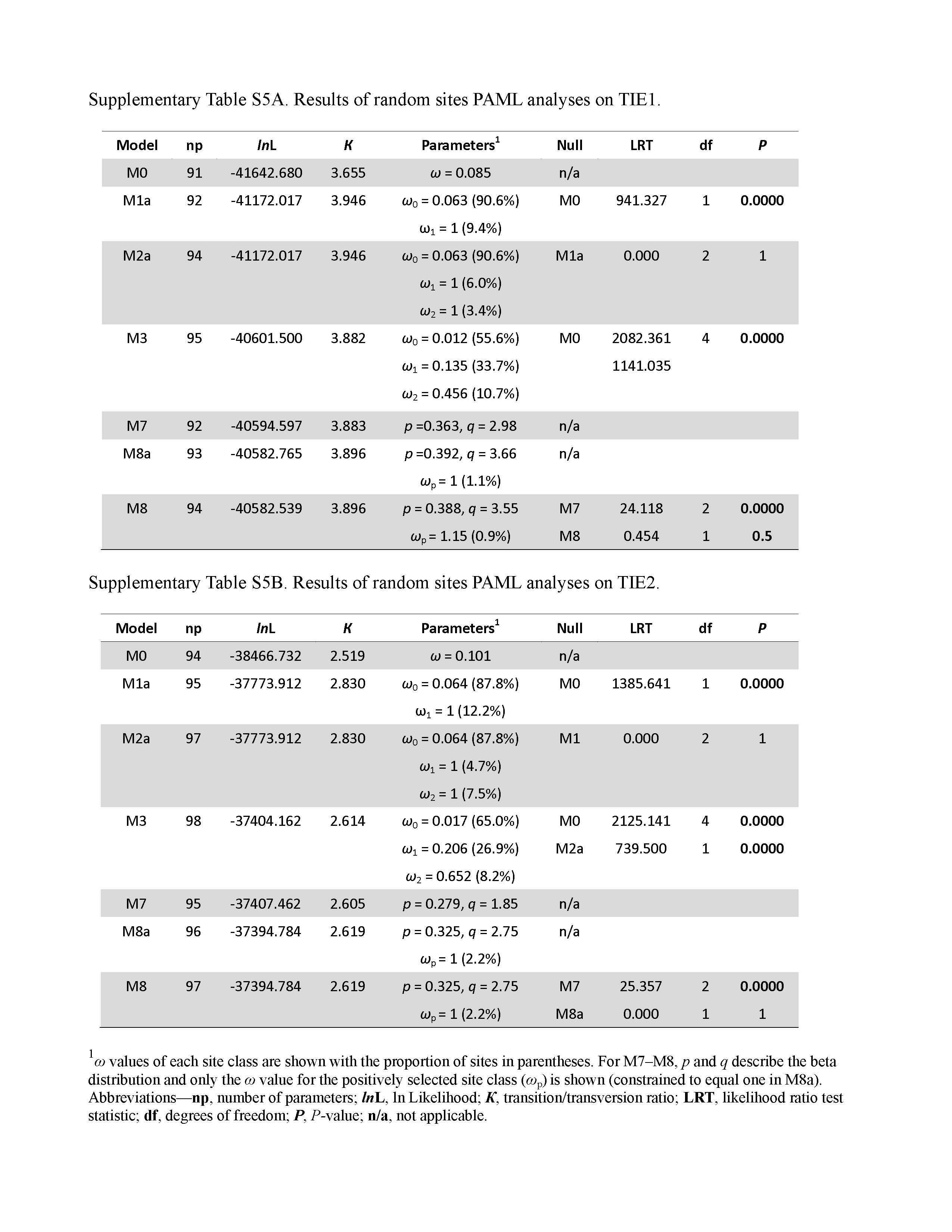

Supplement: Table S5 — Results of random site PAML analysis on (A) TIE1 and (B) TIE2. (TIF) [file pone.0086088.s012.tif]
