# Supplementary material for: Encoding Asymmetry of the N-Glycosylation Motif Facilitates Glycoprotein Evolution
Source: PLoS One. 2014 Jan 24;9(1):e86088. doi: 10.1371/journal.pone.0086088 (PMC3901687; doi:10.1371/journal.pone.0086088)
Supplement: Figure S6 — CD28 gene tree (A) and CTLA4 gene tree (B). Estimated by Bayesian inference (MrBayes), and used for the PAML and HYPHY analyses in. Numbers at the nodes are posterior probabilities. (PDF) [file pone.0086088.s006.pdf]

CD28 gene tree

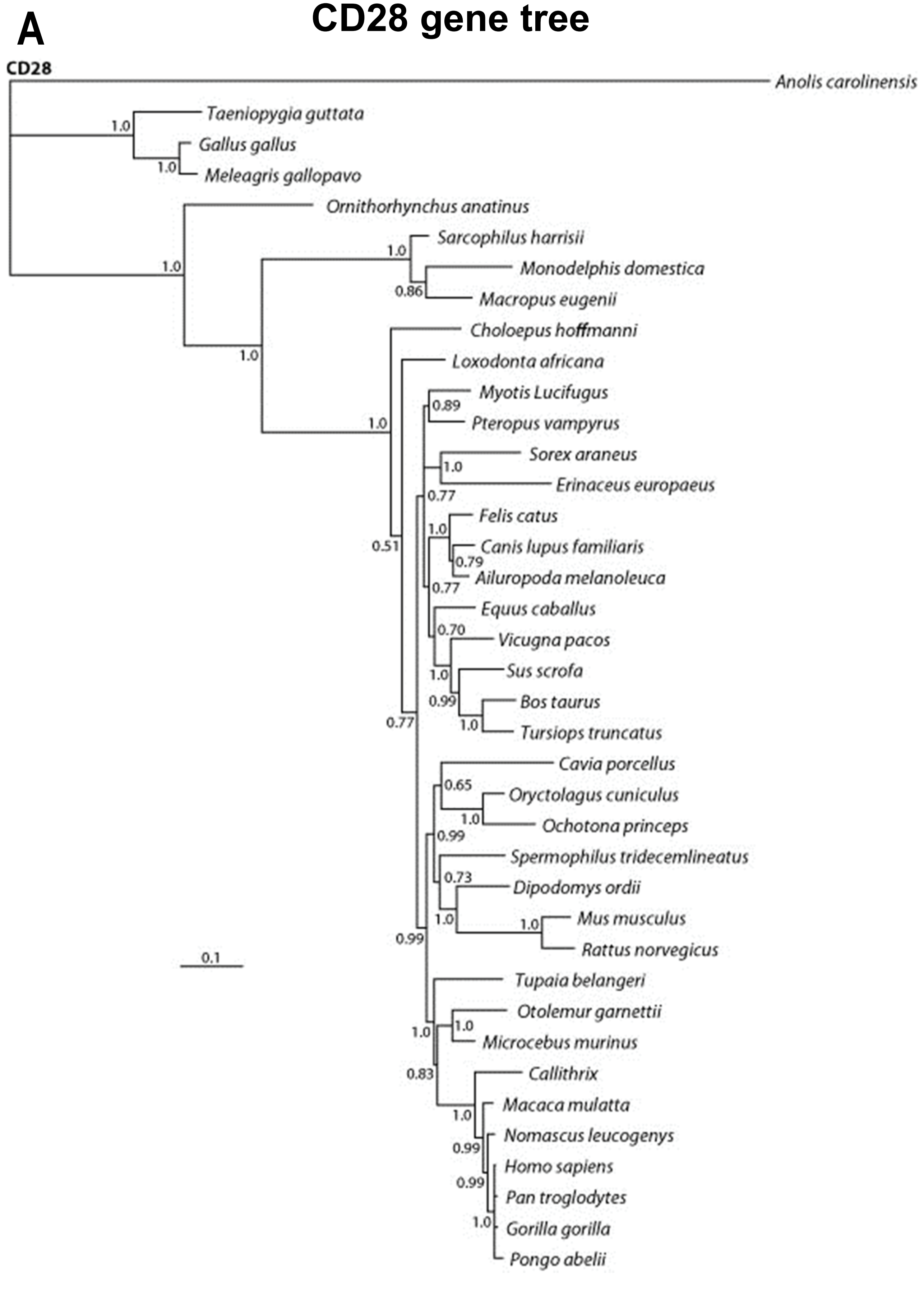

B

CTLA4 gene tree

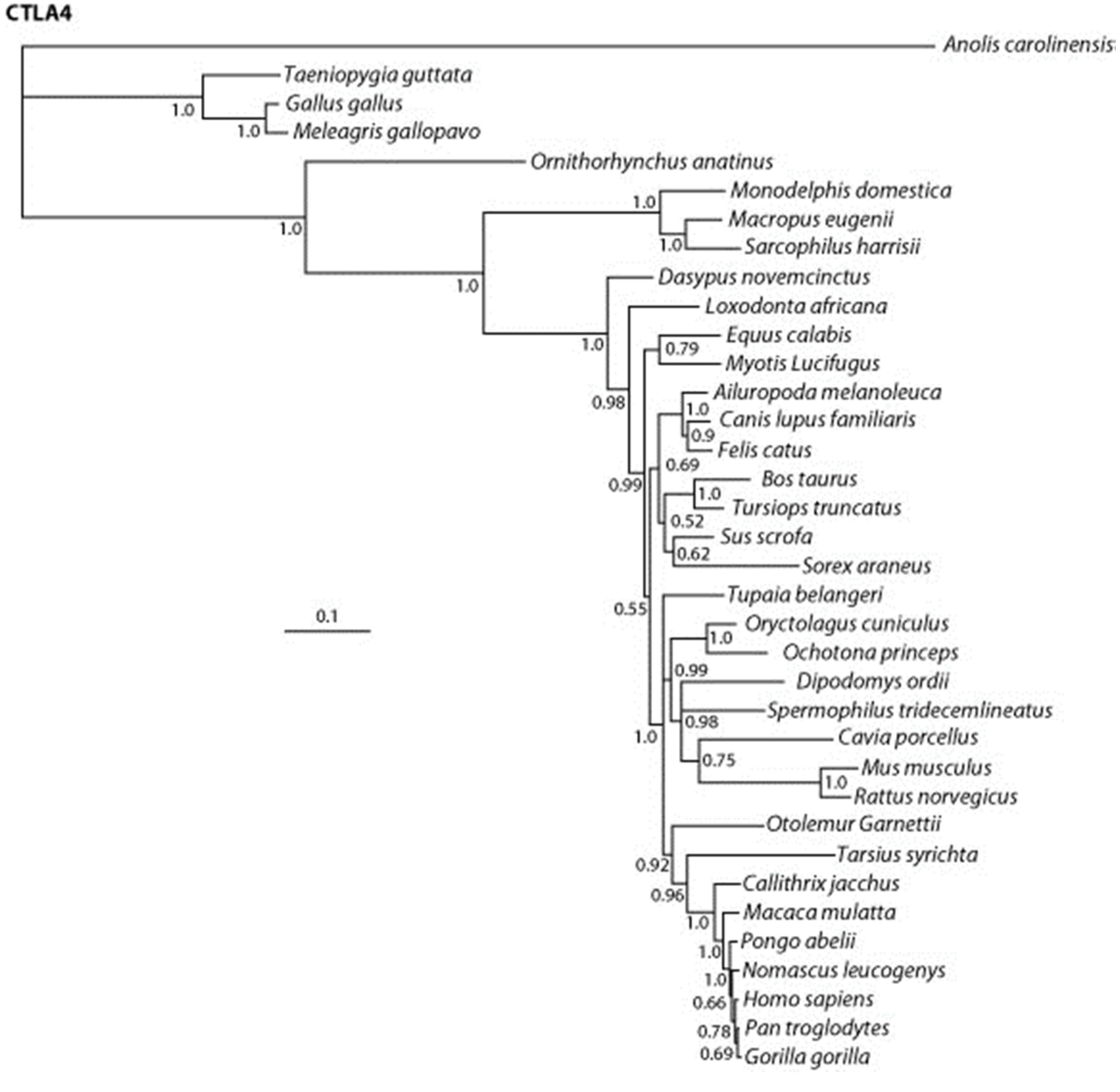

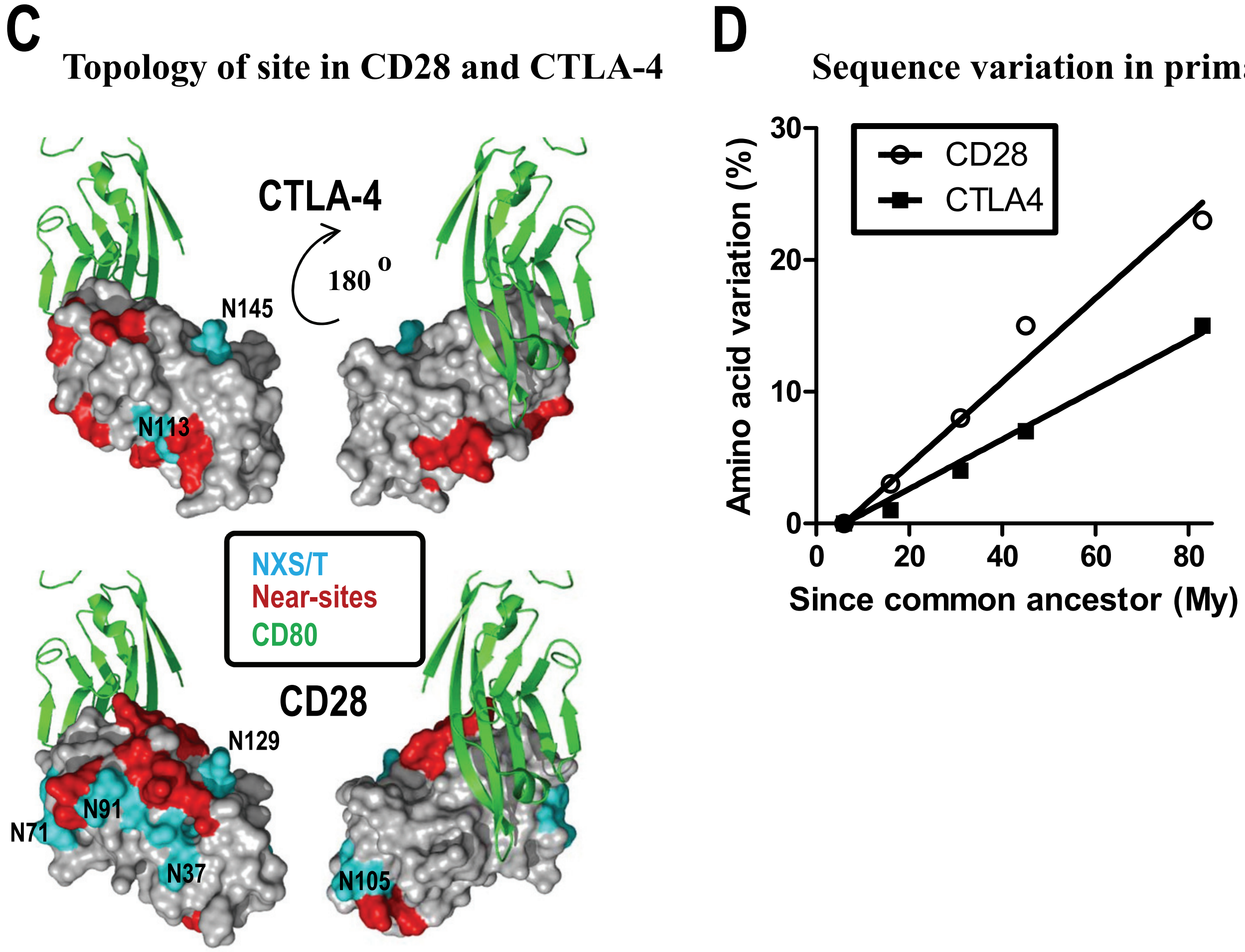
C
Topology of site in CD28 and CTLA-4
CTLA-4
180 o
N145
N113
NXS/T
Near-sites
CD80
CD28
N129
N91
N71
N37
N105
D
Sequence variation in primates
CD28
CTLA4
Amino acid variation (%)
Since common ancestor (My)
0
10
20
30
0
20
40
60
80
