# Supplementary material for: Encoding Asymmetry of the N-Glycosylation Motif Facilitates Glycoprotein Evolution
Source: PLoS One. 2014 Jan 24;9(1):e86088. doi: 10.1371/journal.pone.0086088 (PMC3901687; doi:10.1371/journal.pone.0086088)
Supplement: Table S6 — Clade model C (PAML) analyses on TIE1 where the data was divided into multiple partitions (A) and clade model C (PAML) analyses on TIE2 where the data was divided into eutherian and non-eutherian partitions (B). (PDF) [file pone.0086088.s013.pdf]

Supplementary Table S6A. Results of clade model C (PAML) analyses on TIE1 where the data was divided into multiple partitions.

Model & Partition[1]	np	*ln*L	*K*	Parameters[2]	Null	LRT	df	*P*
M2a	94	-41172.02	3.95	ω_0 = 0.064 (87.8%)<br>ω_1 = 1 (4.7%)<br>ω_2 = 1 (7.5%)	n/a			
M2a_rel	94	-40631.86	3.90	ω_0 = 0.018 (63.9%)<br>ω_1 = 1 (2.5%)<br>ω_2 = 0.211 (33.7%)	n/a			
CmC<br>Mammal	95	-40630.75	3.89	ω_0 = 0.018 (63.0%)<br>ω_1 = 1 (27.8%)<br>Reptiles:<br>ω_d = 0.250 (34.2%)<br>Mammals:<br>ω_d = 0.193 (34.2%)	M2a<br>M2a_rel	1082.53<br>2.22	1<br>1	**0.0000**<br>0.1363
**Therian**	95	**-40627.40**	3.87	ω_0 = 0.018 (63.0%)<br>ω_1 = 1 (2.8%)<br>Non-therians:<br>ω_d = 0.250 (34.2%)<br>Therians:<br>ω_d = 0.193 (34.2%)	M2a<br>M2a_rel	1089.23<br>8.92	1<br>1	**0.0000**<br>**0.0028**
Eutherian	95	-40627.45	3.87	ω_0 = 0.018 (63.2%)<br>ω_1 = 1 (2.8%)<br>Non-eutherians:<br>ω_d = 0.238 (34.1%)<br>Eutherians:<br>ω_d = 0.190 (34.1%)	M2a<br>M2a_rel	1089.14<br>8.83	1<br>1	**0.0000**<br>**0.0030**
Primate	95	-40631.57	3.90	ω_0 = 0.018 (64.0%)<br>ω_1 = 1 (2.4%)<br>Non-primates:<br>ω_d = 0.209 (33.6%)<br>Primates:<br>ω_d = 0.226 (33.6%)	M2a<br>M2a_rel		1<br>1	**0.0000**<br>0.4465

[1]Only the foremost partition is listed. In all cases an additional partition exits that contains the remaining taxa. The best-fitting partition is bolded.
[2]ω values of each site class are shown with the proportion of sites in parentheses. For CmC, ω_d is the divergent site class where the ω value is allowed to differ between the two partitions. Abbreviations—**np**, number of parameters; ***ln*L**, ln Likelihood; ***K***, transition/transversion ratio; **LRT**, likelihood ratio test statistic; **df**, degrees of freedom; ***P***, *P*-value; **n/a**, not applicable.

Supplementary Table S6B. Results of clade model C (PAML) analyses on TIE2 where the data was divided into eutherian and non-eutherian partitions.

Model & Partition[1]	np	*ln*L	*K*	Parameters[1]	Null	LRT	df	*P*
M2a	97	-37773.91	2.83	ω_0 = 0.064 (87.8%)<br>ω_1 = 1 (4.7%)<br>ω_2 = 1 (7.5%)	n/a			
M2a_rel	98	-37415.51	2.64	ω_0 = 0.021 (69.3%)<br>ω_1 = 1 (3.6%)<br>ω_2 = 0.274 (27.1%)	n/a			
CmC<br>Mammal	98	-37388.11	2.63	ω_0 = 0.019 (66.6%)<br>ω_1 = 1 (4.2%)<br>Reptiles:<br>ω_d = 0.429 (29.9%)<br>Mammals:<br>ω_d = 0.217 (29.9%)	M2a<br>M2a_rel	771.61<br>54.81	1<br>1	**0.0000**<br>**0.0000**
Therian	98	-37339.7	2.63	ω_0 = 0.018 (65.3%)<br>ω_1 = 1 (4.7%)<br>Non-therians:<br>ω_d = 0.479 (30.0%)<br>Therians:<br>ω_d = 0.174 (30.0%)	M2a<br>M2a_rel	868.42<br>151.62	1<br>1	**0.0000**<br>**0.0000**
**Eutherian**	98	**-37330.86**	2.63	ω_0 = 0.017 (65.1%)<br>ω_1 = 1 (4.8%)<br>Non-eutherians:<br>ω_d = 0.448 (30.1%)<br>Eutherians:<br>ω_d = 0.161 (30.1%)	M2a<br>M2a_rel	886.09<br>169.30	1<br>1	**0.0000**<br>**0.0000**
Primate	98	-37414.64	2.64	ω_0 = 0.276 (26.8%)<br>ω_1 = 1 (3.6%)<br>Non-primates:<br>ω_d = 0.022 (69.6%)<br>Primates:<br>ω_d = 0.016 (69.6%)	M2a<br>M2a_rel	718.54<br>1.75	1<br>1	**0.0000**<br>0.1861

[1]Only the foremost partition is listed. In all cases an additional partition exits that contains the remaining taxa. The best-fitting partition is bolded.

[2]ω values of each site class are shown with the proportion of sites in parentheses. For CmC, ω_d is the divergent site class where the ω value is allowed to differ between the two partitions. Abbreviations—**np**, number of parameters; ***ln*L**, ln Likelihood; ***K***, transition/transversion ratio; **LRT**, likelihood ratio test statistic; **df**, degrees of freedom; ***P***, *P*-value; **n/a**, not applicable.
